# Characterization of HIV diversity, phylodynamics and drug resistance in Washington, DC

**DOI:** 10.1371/journal.pone.0185644

**Published:** 2017-09-29

**Authors:** Marcos Pérez-Losada, Amanda D. Castel, Brittany Lewis, Michael Kharfen, Charles P. Cartwright, Bruce Huang, Taylor Maxwell, Alan E. Greenberg, Keith A. Crandall, on behalf of the DC Cohort Executive Committee

**Affiliations:** 1 Computational Biology Institute, Milken Institute School of Public Health, The George Washington University, Ashburn, VA, United States of America; 2 CIBIO-InBIO, Universidade do Porto, Campus Agrário de Vairão, Vairão, Portugal; 3 Milken Institute School of Public Health, Department of Epidemiology and Biostatistics, The George Washington University, Washington, DC, United States of America; 4 District of Columbia Department of Health, Washington, DC, United States of America; 5 Laboratory Corporation of America, Burlington, NC, United States of America; National and Kapodistrian University of Athens, GREECE

## Abstract

**Background:**

Washington DC has a high burden of HIV with a 2.0% HIV prevalence. The city is a national and international hub potentially containing a broad diversity of HIV variants; yet few sequences from DC are available on GenBank to assess the evolutionary history of HIV in the US capital. Towards this general goal, here we analyze extensive sequence data and investigate HIV diversity, phylodynamics, and drug resistant mutations (DRM) in DC.

**Methods:**

Molecular HIV-1 sequences were collected from participants infected through 2015 as part of the DC Cohort, a longitudinal observational study of HIV+ patients receiving care at 13 DC clinics. Sequences were paired with Cohort demographic, risk, and clinical data and analyzed using maximum likelihood, Bayesian and coalescent approaches of phylogenetic, network and population genetic inference. We analyzed 601 sequences from 223 participants for *int* (~864 bp) and 2,810 sequences from 1,659 participants for *PR/RT* (~1497 bp).

**Results:**

Ninety-nine and 94% of the *int* and *PR/RT* sequences, respectively, were identified as subtype B, with 14 non-B subtypes also detected. Phylodynamic analyses of US born infected individuals showed that HIV population size varied little over time with no significant decline in diversity. Phylogenetic analyses grouped 13.5% of the *int* sequences into 14 clusters of 2 or 3 sequences, and 39.0% of the *PR/RT* sequences into 203 clusters of 2–32 sequences. Network analyses grouped 3.6% of the *int* sequences into 4 clusters of 2 sequences, and 10.6% of the *PR/RT* sequences into 76 clusters of 2–7 sequences. All network clusters were detected in our phylogenetic analyses. Higher proportions of clustered sequences were found in zip codes where HIV prevalence is highest (r = 0.607; P<0.00001). We detected a high prevalence of DRM for both *int* (17.1%) and *PR/RT* (39.1%), but only 8 *int* and 12 *PR/RT* amino acids were identified as under adaptive selection. We observed a significant (P<0.0001) association between main risk factors (men who have sex with men and heterosexuals) and genotypes in the five well-supported clusters with sufficient sample size for testing.

**Discussion:**

Pairing molecular data with clinical and demographic data provided novel insights into HIV population dynamics in Washington, DC. Identification of populations and geographic locations where clustering occurs can inform and complement active surveillance efforts to interrupt HIV transmission.

## Introduction

Washington, DC has consistently had one of the highest rates of HIV infection in the United States (US) with 371 new HIV cases reported in 2015 and a 2.0% HIV prevalence [1]. The HIV epidemic in DC is generalized: seven of the city’s eight wards (geopolitical areas) have an HIV prevalence greater than one percent and men who have sex with men (MSM) and heterosexuals (HRH) account for 45% and 28% respectively of new diagnoses, with those of unknown risk accounting for 22%. The other 3% and 2% are IDU and sexual contact/IDU, respectively [1]. Measurement of the DC HIV continuum of care finds that approximately 11% of people living with HIV infection (PLWH) are estimated to be unaware of their infections [2] and only 73% of persons are continuously in care with 57% of all PLWH achieving viral suppression as of last report [1]. Given that a relatively high proportion of PLWH in care in DC have detectable viral loads (VL) [3] and that PLWH with VL of 1,500 copies/ml or higher are at increased risk for transmitting virus [4], identifying those individuals is critical to ensure they are receiving appropriate antiretroviral therapy and to curbing new infections. Furthermore, DC is a national and international city potentially containing a broad diversity of HIV variants with 11% of PLWH in DC being foreign-born [5]. Previous phylogenetic studies in the DC-Maryland region have found that 13% of people are infected with non-B-subtypes and of those, 81% were from the Maryland suburbs of DC [6]. Thus, further characterization of subtype distribution will assist in determining whether there are distinct HIV epidemics occurring in the region [6].

Started in 2011, the DC Cohort is a longitudinal observational cohort study that aims to characterize the quality of care being received among PLWH obtaining outpatient care at 13 clinic sites in DC. With over 8,000 participants enrolled as of December 2016, the Cohort provides a representative sample of the approximately 13,000 PLWH in DC who are in care [1]. The DC Cohort demographics are similar to those of all PLWH with respect to age, race/ethnicity, and sex; however, it is important to note that Cohort participants are those in care and may not reflect the care patterns of all PLWH in DC. All consented participants have de-identified data electronically exported monthly to a centralized database [7]. Data elements abstracted from sites’ electronic medical records (EMRs) include demographics, HIV risk behaviors, diagnoses, laboratory tests, treatments, and procedures. An important element of the DC Cohort includes the periodic linkage of Cohort data to the DC Department of Health (DOH) HIV/AIDS Hepatitis, STD, TB Administration (HAHSTA) surveillance databases inclusive of molecular sequences from commercial laboratory reports, as well as location information such as zip code of residence. In this study, GW, DC DOH and LabCorp collaborated to further analyze these sequences beyond individual drug resistant variant calling–the only information being currently reported back to DOH from LabCorp. The objectives of these analyses were to: 1) characterize the diversity of HIV in the recent history of the DC epidemic, 2) identify drug resistant variants and sites under natural selection circulating in the DC population, 3) identify potential transmission networks, and 4) characterize associations of epidemiological factors with potential transmission networks, including geography.

## Materials and methods

### Ethics

Institutional Review Board (IRB071029) approval was obtained from The George Washington University IRB (which serves as the IRB of Record for eight of the participating sites), the DC DOH IRB, and the remaining site IRBs. Written informed consent was obtained and documented prior to conducting study procedures.

### DC cohort

The DC DOH is one of 25 US jurisdictions funded by the Centers for Disease Control and Prevention to conduct Molecular HIV Surveillance [8]. Through this surveillance program, molecular sequence data generated by commercial laboratories is routinely sent to the DC DOH and incorporated into the surveillance database. For the purposes of this analysis, resistance data generated by LabCorp and sent to the DC DOH was incorporated into the DC Cohort database through the aforementioned linkage process. Between January 1, 2011 and March 31, 2015, 6,800 DC Cohort participants were enrolled in the study. The DC DOH received 3,411 sequences collected between 2011 and 2015 on DC Cohort participants, which were collected from 1,895 unique individuals. Using the most recently available post-DOH linked data for participants consented by March 31, 2015 and sequenced by June 15, 2015, 2,858 sequences were available representing 1,740 unique participants (i.e., sequence coverage is 25.6%). Descriptive and univariate analyses using Chi-square and Wilcoxon rank sum tests in SAS v9.3 were conducted to describe participants and examine differences between Cohort participants with and without sequence data (Table 1). The PROC FREQ function was used to perform the Chi-Square tests and PROC NPAR1WAY function was used to perform the Wilcoxon rank sum test. The sequence data were merged by participant ID with a limited set of DC Cohort demographic and clinical variables (e.g., age, race, sex, risk factor, CD4 and viral load count). The paired data were then analyzed in a de-identified manner using approaches described below.

**Table 1:** Demographic and clinical characteristics of DC Cohort participants stratified by availability of sequence data.

	Total N^1^ = 6,800	Participants Sequenced N^1^ = 1,740	Participants not Sequences N^1^ = 5,060	p-value^2^
**Median Age (IQR)**^**3**^	47 (36.4,54.6)	43 (31.6,50.7)	48.3 (38.5,55.7)	< .0001
**Race/ethnicity**^**3**^				
Non-Hispanic Black	5,317 (78.3)	1,486 (85.4)	3,831 (75.8)	
Non-Hispanic White	982 (14.5)	148 (8.5)	834 (16.5)	
Hispanic	334 (4.9)	77 (4.4)	257 (5.1)	< .0001
Other	146 (2.1)	26 (1.5)	120 (2.4)	
Unknown	12 (0.2)	3 (0.2)	9 (0.2)	
**Sex at Birth**^**3**^				
Male	4,938 (72.6)	1,162 (66.8)	3,776 (74.6)	
Female	1,862 (27.4)	578 (33.2)	1,284 (25.4)	< .0001
**Country of Birth**^**3**^				
US	1,117 (82.6)	128 (75.3)	989 (83.7)	
Non-US	235 (17.4)	42 (24.7)	193 (16.3)	.0070
**State of Residence**^**3**^				
DC	4,994 (73.4)	1,553 (89.2)	3,441 (68.0)	
MD	1,298 (19.1)	155 (8.9)	1,143 (22.6)	< .0001
VA	406 (6.0)	29 (1.7)	377 (7.4)	
Other	102 (1.5)	3 (0.2)	99 (2.0)	
**HIV Risk Factor**^**3**^				
MSM	3,272 (48.1)	844 (48.5)	2,428 (48.0)	
MSM/IDU	110 (1.6)	26 (1.5)	84 (1.6)	
IDU	959 (14.1)	234 (13.4)	725 (14.3)	< .0001
Heterosexual	1,961 (28.8)	555 (31.9)	1,406 (27.8)	
Other^4^	262 (3.8)	39 (2.2)	223 (4.1)	
Unknown	236 (3.5)	42 (2.4)	194 (3.8)	
**Co-morbidities**^**3**^				
Hepatitis C	820 (12.1)	115 (6.6)	705 (13.9)	< .0001
Hepatitis B	235 (3.5)	41 (2.4)	194 (3.8)	0.0036
Syphilis	260 (3.8)	84 (4.8)	176 (3.5)	0.0113
Gonorrhea	40 (0.6)	25 (1.4)	15 (0.3)	< .0001
Chlamydia	40 (0.6)	16 (0.9)	24 (0.5)	0.0362
**Median Duration of Infection (yrs)(IQR)**^**3**^	10 (5,17)	8 (2,15)	11 (6,17)	< .0001
**Median CD4 count (cells/μl)(IQR)**^**3**^	513 (323,723)	348.5 (171,534)	566 (391.5,772)	< .0001
**Viral Load (copies/ml) (IQR)**^**3**^				
<200	3109 (59.9)	88 (5.9)	3,021 (81.9)	
200–399	159 (3.1)	46 (3.1)	113 (3.1)	
400–9999	733 (14.1)	473 (31.6)	260 (7.1)	< .0001
≥10,000	1188 (22.8)	892 (59.5)	296 (8.0)	
**ARV Exposure**^**3**^				
Experienced	5,905 (86.8)	1,171 (67.3)	4,734 (93.6)	
Naïve	345 (5.1)	117 (6.7)	228 (4.5)	< .0001
Unknown	550 (8.1)	452 (26)	98 (1.9)	
**ARV Regimen Type**^**3**^				
PI-based	145 (2.4)	80 (1.8)	65 (4.2)	
NRTI-based	234 (3.8)	152 (3.3)	82 (5.3)	
NNRTI—based	13 (4.3)	7 (0.1)	6 (0.4)	< .0001
INSTI-based	56 (0.9)	42 (0.9)	14 (0.9)	
Dual-Class	3,069 (50.4)	2,270 (50.1)	799 (51.3)	
**ARV Resistance**^**3**^				
At least one PI	874 (13.9)	210 (7.9)	664 (18.3)	< .0001
At least one NRTI	1,687 (26.8)	537 (20.2)	1,150 (31.6)	< .0001
At least one NNRTI	1,645 (26.1)	660 (24.8)	985 (27.1)	0.0446
Other	24 (0.4)	19 (0.7)	5 (0.1)	0.0002

^1^Represents total number of Cohort participants enrolled through March 31, 2015. Totals may not sum to *N* due to missing data

^2^Chi-square or Wilcoxon test

^3^Data at the time of first sequence

^4^Perinatal, blood transfusion, hemophilia/coagulation disorder

### Sequencing

Sequencing-based analyses of regions of the HIV-1 polymerase (*pol*) gene were performed by LabCorp for the detection of anti-retroviral resistance polymorphisms in regions encoding the protease (PR; codons 1–99), reverse transcriptase (RT; codons 1–400), and integrase (INT; codons 1–288) coding regions. In brief, HIV-1 RNA was recovered from plasma samples and reverse transcription followed by nested polymerase-chain-reaction (RT-PCR) performed using HIV-1 specific primer sets. Upon successful amplification, products were subjected to Sanger sequencing (BigDye® Terminator v3.1, ThermoFisher) and sequence reads analyzed using Sequencher DNA Sequence Analysis Software (Gene Codes Corp.). This process resulted in the generation of two independent contiguous sequences of 1497bp of *PR/RT* (corresponding to nucleotide positions 2253–3749 of HBX2CG [GenBank accession K03455]) and 864bp *int* (corresponding to nucleotide positions 4230–5093 of HBX2CG).

### Analyses

Sequence data were collected from HIV positive plasma samples from DC Cohort individuals targeting either a portion of the *PR/RT* and/or *int*. We also included 170 subtype reference sequences from the Los Alamos HIV database (http://www.hiv.lanl.gov/) to assign sequences to particular subtype clades. Sequence data were aligned using MAFFT [9]. The best-fit model of molecular evolution [10] was estimated from the data using jModelTest [11]. A maximum likelihood phylogenetic estimate [12] was made using RAxML and 3 codon-position partitions [13] with the best-fit model for each partition. Nodal support was estimated using the bootstrap approach with 1,000 replicates [14]. Bayesian trees were also inferred using MrBayes [15] and 3 codon-position partitions. We ran four chains (one cold and three heated) for 4x10^7^ generations sampling every 2,000 steps for the *int* region and for 10^8^ generations sampling every 4,000 steps for the *PR/RT* region. Each run was repeated twice. Convergence and mixing of the Markov chains were assessed in Tracer [16]. Phylogenetic transmission (infection) clusters [17] were defined as those clades with bootstrap proportions >70% and posterior probabilities >0.95. Transmission chains were also assessed using a recently described network approach [18, 19] implemented in HIV-Trace (http://test.datamonkey.org/hivtrace). We used genetic distance thresholds of 0.01 substitutions/site for identifying potential transmission partners (see [18]).

HIV subtype B relative genetic diversity (i.e., population size over time) was inferred in BEAST [20] using the *PR/RT* sequence data for all infected individuals born in the US. We assume that these patients were also infected in the US. No sequence data were available for *int* from US born patients. We used the GMRF Bayesian Skyride model [21], the HKY substitution model with gamma-distributed among-site rate heterogeneity, and a relaxed clock (lognormal) model of rate of substitution [22]. We used the date of HIV-1 diagnosis to calibrate the analysis and a normal prior with mean = 0.001 and SD = 0.0005 for ucld.mean. We performed two runs 2x10^7^ generations long sampling every 1,000 generations. Parameter uncertainty was summarized in the 95% highest posterior density (HPD) intervals. All output generated by BEAST was analyzed in Tracer [23].

HIV-1 subtype identification was done using the REGA subtyping tool [24, 25] and validated via phylogenetic analyses above. Haplotype diversity (h), the number of segregating sites (S), nucleotide diversity (π), Watterson genetic diversity (θ) and recombination rate (r) were estimated using DnaSP v. 5.10.1 [26]. Nucleotide ambiguities in the alignment were arbitrarily resolved for these analyses. We identified drug resistant mutations by BLASTing [27] nucleotide sequences against the Stanford HIV Drug Resistance Database (https://hivdb.stanford.edu) using the HIVdb Program. We then identified nucleotide positions under positive selection using Fast Unconstrained Bayesian AppRoximation (FUBAR) [28], while accounting for recombination GARD [29, 30]. These analyses were carried out in HyPhy [31].

Because the HIV sequences are related through an hierarchical evolutionary history, they are not independent and typical genotype to phenotype associations cannot be performed without taking the dependence structure into account [32]. We intended to use treescanning [33] for the analyses, but found that there was insufficient resolvable structure for *int* data set and although there were several large clades for *PR/RT*, there was no resolution between them at the base of the tree (i.e., low bootstrap values and posterior probabilities for clade structure among these large clades). Therefore, among the few clades large enough for statistical inference, we treated each clade as independent and conducted simple contingency table tests for association with sex, race and risk behavior where each clade was treated as a separate factor and the remaining sequences were grouped into one factor using a permutation chi-square test [34]. We used the chisq.test function in the core “stats” package of R [35] with the simulate.p.value option. We generated 100 million Monte Carlo permutations [36] for each test to obtain empirical p-values. To increase the number of cases per cell in our chi-square tests, we limited the risk category to the two main risk types (HRH, IDU and MSM). Because MSM and HRH are highly correlated with sex, we also analyzed gender excluding MSM.

## Results

### Samples

We paired sequence data with the most recently available post-DOH linked demographic and clinical data for participants consented by March 31, 2015 and sequenced by June 15, 2015. We collected 601 partial *int* sequences and 2,810 partial *PR/RT* sequences from 223 participants and 1,659 participants, respectively, with a few participants being sequenced for both genes. All sequences have been deposited in GenBank under accession numbers MF455515 –MF457397. Aggregated demographic and clinical information (race, sex, age, risk factor, viral load, CD4+ count, sequence date, HIV diagnosis date, and zip code) for participants are shown in Table 1 along with a comparison between DC Cohort participants who had sequences available to those without sequences. Our data came from participants who resided predominantly in Washington DC (73.4%) and had dominant HIV risk factors of MSM (48.1%) and heterosexual (28.8%). Participants with sequence availability were significantly more likely to be younger, black, female, non-US born, and DC residents (p<0.0001 for all) compared to those without sequence data. Slightly more participants with sequences were infected through heterosexual sexual contact, and a higher proportion had a history of hepatitis C, syphilis, gonorrhea, and chlamydia co-infections at the time of sequencing (p<0.05 for all). Participants with sequences had a shorter duration of infection, lower CD4 counts, higher viral loads, and were less likely to be treatment experienced (p<0.0001 for all). Among those on ARVs, a higher proportion of those sequenced were on dual-class regimens and had a lower prevalence of resistance to PIs, NRTIs, and NNRTIs (p<0.001 for all).

### HIV-1 diversity

**Fig 1:**
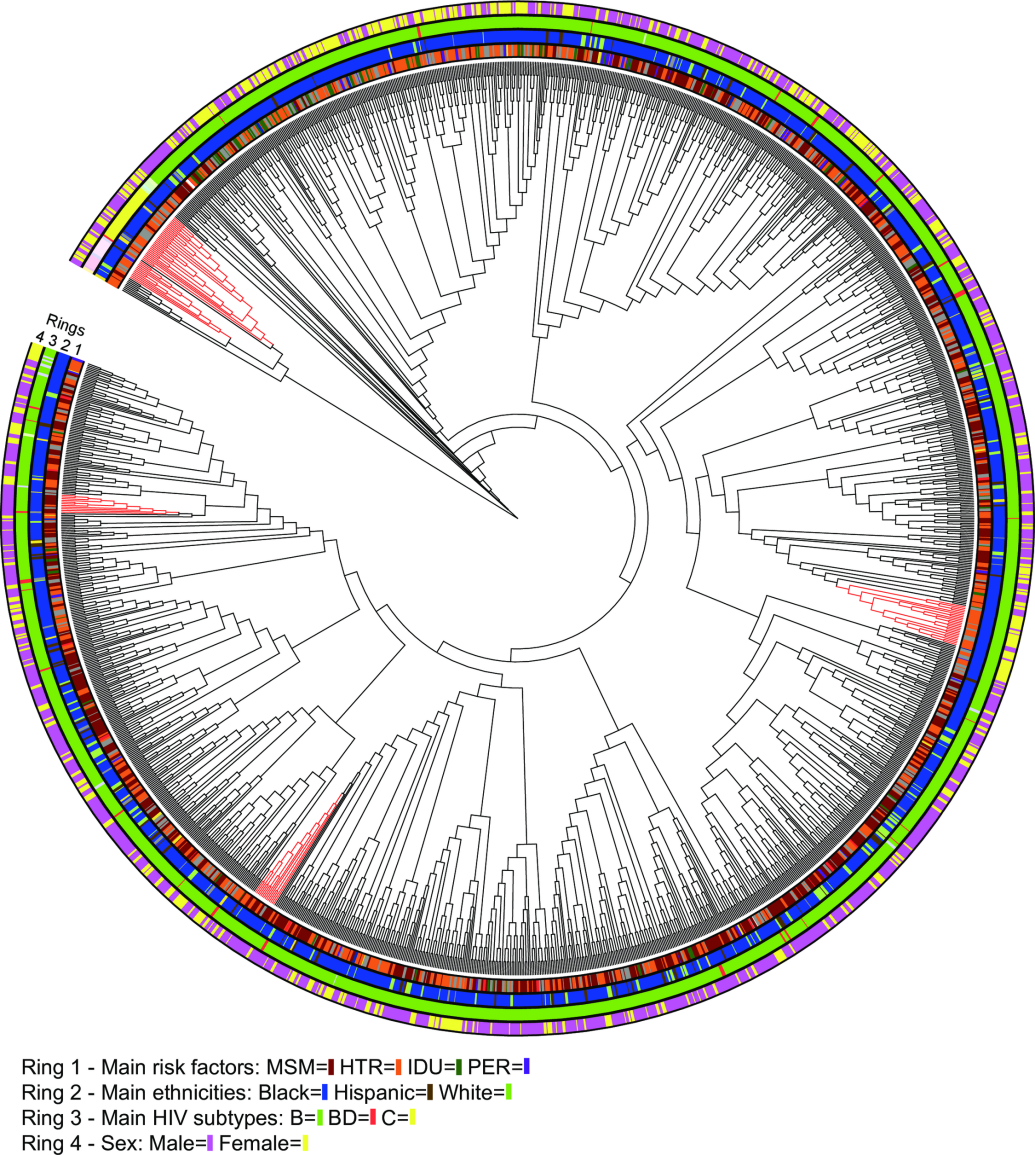
Cladogram of the *PR/RT* gene showing risk factors, ethnicities, subtypes and sex in four concentric rings. Main phenotypes within each ring are represented with different colors. Well-supported clades comprised of >10 HIV sequences are also indicated in red.

Our subtyping analysis of *int* found that 220 of the participants were infected with subtype B virus and identified three additional subtypes: A (1 individual), C (1), and G (1). The *PR/RT* sequences were also heavily subtype B (1,557 of 1,659 participants), but with a greater diversity of other subtypes including: AG (9 individuals), CD (1), BF (2), A (7), C (32), D (4), F (1), G (3), J (1), AK (1), BA (1), BD (29), BF (7), DB (1) and unknown (3) (see also Fig 1).

All the DNA sequences from the same individual comprised identical replicates, hence we only used one representative per individual for our analyses. The subtype B *PR/RT* gene showed higher diversity rates, substitution rates (estimated as in [37]) and minimum number of recombinant events than the subtype B *int* gene, but lower recombination/gen rates (Table 2). Watterson genetic diversity (θ) for subtype B *int* was lower in the IDU group compared to subtype B HRH and MSM likely due to the smaller sample size of the IDU (Table 2). For the subtype B *PR/RT* sequence data, we also included perinatally infected participants and that group had the highest nucleotide diversity, θ and non-synonymous substitution rates despite its smaller sample size.

**Table 2:** HIV DNA polymorphism and drug resistant mutations (DRM).

	Diversity					Substitutions		Recombination		DRM
	N	S	h	Pi	θ (W)	Pi(s)	Pi(ns)	R/gene	Rm	Total (relative %)
**SubB**										
*Int*										
ALL	220	434	220	0.054	0.084	0.188	0.024	1133	100	38 (17.3)
HRH	78	345	78	0.053	0.081	0.185	0.023	831	83	2 (2.6)
IDU	13	180	13	0.051	0.067	0.174	0.024	3134	37	3 (23.1)
MSM	71	350	71	0.054	0.084	0.190	0.024	1178	85	20 (28.2)
*PR/RT*										
ALL	1557	720	1556	0.064	0.090	0.222	0.028	975	149	584 (39.1)
HRH	512	614	512	0.063	0.088	0.220	0.028	1210	141	215 (38.5)
IDU	85	456	85	0.055	0.089	0.187	0.024	348	111	28 (32.6)
MSM	605	636	604	0.065	0.090	0.228	0.028	1304	143	232 (36.4)
PER	37	424	37	0.070	0.099	0.215	0.039	385	101	26 (68.4)
**SubBD**										
*PR/RT*										
ALL	29	353	29	0.067	0.088	0.239	0.029	2872	89	10 (34.5)
HRH	8	199	8	0.066	0.075	0.231	0.030	4363	37	1 (3.4)
MSM	18	294	18	0.067	0.083	0.243	0.028	1293	61	8 (27.6)
**SubC**										
*PR/RT*										
ALL	32	354	32	0.066	0.086	0.243	0.027	957	91	8(25.0)
HRH	16	273	16	0.066	0.080	0.236	0.029	1895	69	3 (9.4)

Diversity (N = sequences, S = segregating sites, h = haplotypes, Pi = nucleotide diversity, θ = Watterson genetic diversity), substitutions (Pi(s) = synonymous, non-synonymous Pi(ns)), recombination (R) (R/gen = R per gen, and Rm = minimum number of recombinant events) rates. Total and relative (total/N) proportion (%) of HIV strains including DRM. MSM = Men who have sex with men, HRH = heterosexuals, IDU = intravenous drug users, PER = perinatal

No noticeable differences in diversity and substitution rates were observed among subtypes B, BD, and C for all the indices compared except for recombination per gene, which was about three times higher for subtype BD compared to the other two subtypes.

### Drug resistance

A total of 38 subtype B *int* sequences (2 HRH, 3 IDU and 20 MSM, and 13 “other risk”) showed at least one DRM with an overall prevalence (HIV sequences including at least one DRM/total number of sequences) of 17.3%. Thirty-five IN Major mutations and 26 IN Accessory mutations were detected (Table 3). A total of 584 subtype B *PR/RT* sequences (215 HRH, 28 IDU, 232 MSM, 26 PER and 83 “other risk”) showed at least one DRM with a prevalence of 39.1%. NRTI, NNRTI and RT SDRMs included the highest proportions of resistant individuals (301 to 461) and total DRM (557 to 931) and unique DRM (59 to 80) (Table 3). A total of 10 subtype BD *PR/RT* sequences (1 HRH, 8 MSM and 1 “other risk”) showed at least one DRM with a prevalence of 34.5%. NRTI, NNRTI and RT SDRMs also included the highest proportions of resistant individuals (6 to 10) and total DRM (10 to 22) and unique DRM (7 to 14) (Table 3). Finally, a total of 8 subtype C *PR/RT* sequences (3 HRH and 5 “other risk”) showed at least one DRM with a prevalence of 25.0%. NNRTI and RT SDRMs included the highest proportions of resistant individuals (7) and total DRM (12 to 13) and unique DRM (9 to 12) (Table 3). It is important to highlight that since only 6.7% of the patients in our cohort are treatment naïve, we cannot confirm that these DRM are actually being transmitted. Amino acid mutations counted as a DRM for each subtype and gene region are also presented in S1 Table.

**Table 3:** Drug resistant mutations.

	Sub B – *int*			Sub B – *PR/RT*			Sub BD – *PR/RT*			Sub C – *PR/RT*		
	S	TM	UM	S	TM	UM	S	TM	UM	S	TM	UM
IN Major	24	35	17	-	-	-	-	-	-	-	-	-
IN Accessory	25	26	6	-	-	-	-	-	-	-	-	-
PR Major	-	-	-	87	154	31	0	0	0	1	1	1
PR Accessory	-	-	-	72	107	22	0	0	0	2	2	2
NRTI	-	-	-	301	557	80	6	13	9	3	4	4
NNRTI	-	-	-	410	663	69	6	10	7	7	13	12
PR SDRMs	-	-	-	90	186	26	0	0	0	1	1	1
RT SDRMs	-	-	-	461	931	59	10	22	14	7	12	9
PI TSMs	-	-	-	33	34	11	0	0	0	1	1	1
NRTI TSMs	-	-	-	52	59	13	1	1	1	1	1	1
NNRTI TSMs				14	15	12	1	1	1	0	0	0
DRM Codons		11			72			21			22	
FUBAR Codons		8			14			2			5	

Sequences (S), Total Mutations (TM) and Unique Mutations (UM) conferring resistance to antiretroviral drugs (IN Major to NNRTI TSMs) for genes int and PR/RT in HIV subtypes (Sub) B, BD and C. DRM amino acid codons and codons under adaptive selection (FUBAR) are also listed

Subtype B *int* DRM caused amino acid changes in 11 different codons while Subtype B *PR/RT* DRM caused amino acid changes in 72 different codons (Table 3). Similarly, subtype BD and C *PR/RT* DRM caused amino acid changes in 13 and 19 different codons, respectively. These codons did not correspond to those inferred by FUBAR as being positively selected (8 and 12 amino acids in the *int* and *PR/RT* genes, respectively), except codon 140 in *PR/RT*, which matched in both analyses. Positively selected sites included 8 amino acids for Subtype B *int* (72, 201, 206, 218, 227, 230, 265 and 283), 14 for Subtype B *PR/RT* (12, 13, 19, 35, 37, 59, 79, 95, 136, 140, 203, 236, 301 and 312), 2 for Subtype BD *PR/RT* (136 and 312) and 5 for Subtype C *PR/RT* (19, 65, 236, 274 and 275).

### Phylodynamics and transmission networks

**Fig 2:**
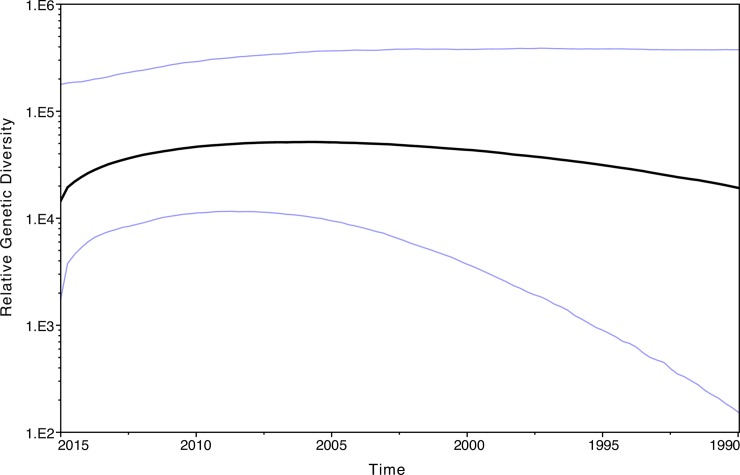
Bayesian skyride plot of HIV-1 subtype B *PR/RT* past population dynamics in US born patients. Black lines show the median estimate and blue lines the 95% high posterior density limits of the relative genetic diversity over time.

**Fig 3:**
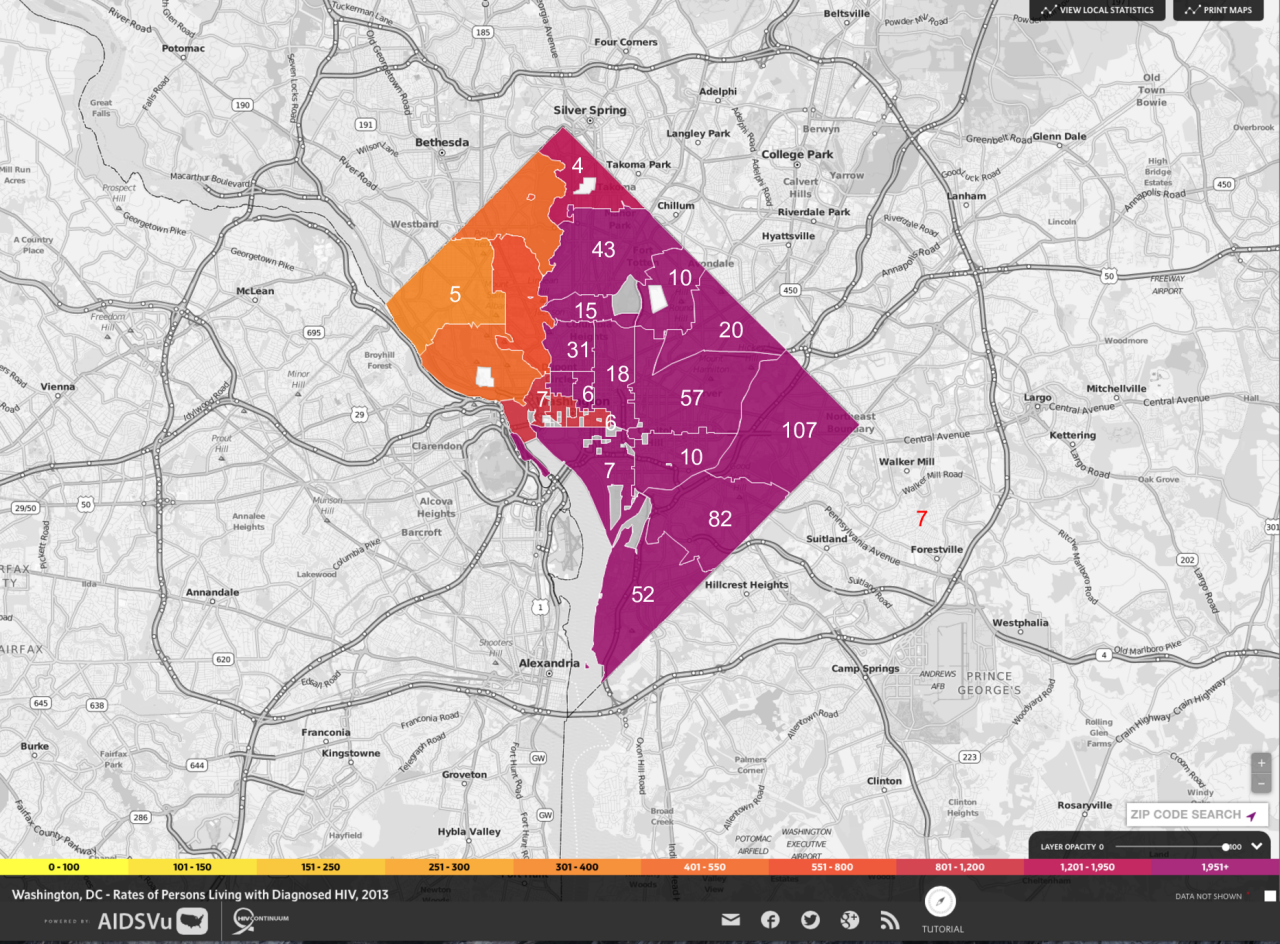
Geographic distribution of transmission networks by zip code overlaid on Washington DC map with rates of persons living with diagnosed HIV in DC. Only zip codes containing ≥4 HIV sequences that fall into a ML cluster (70% bootstrap support) are shown.

**Fig 4:**
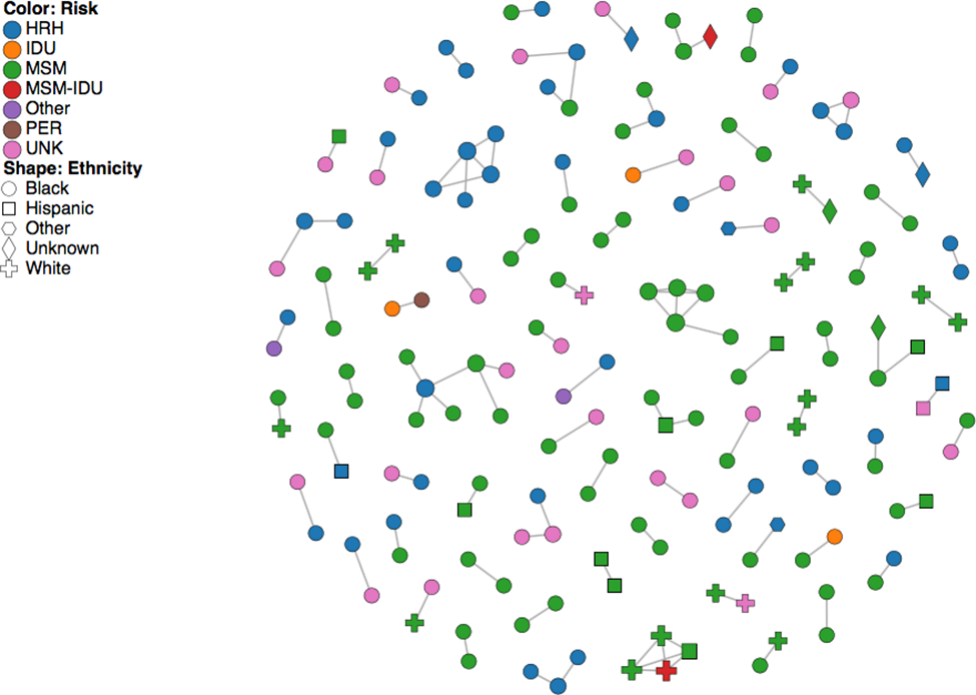
*PR/RT* network (HIV-Trace) of Washington DC HIV-1 isolates showing risk factors and ethnicities.

The past demographic analysis of DC subtype B *PR/RT* sequences from US born patients in BEAST (Fig 2) indicates that HIV relative genetic diversity has not decreased significantly over the last 25 years, despite an increasing prevalence of HIV infection earlier on in the epidemic followed by more recent decreases in HIV incidence rates [1, 38]. Additionally BEAST analyses aggregating HIV sequences by the two main sexual orientations of their hosts (MSM and HTR) generated similar Skyride plots to that presented in Fig 2. Our ML phylogenies and bootstrap analysis (70% support) grouped 13.5% of the *int* sequences into 14 phylogenetic clusters comprised of 2 to 3 sequences, while 39.0% of the *PR/RT* sequences grouped into 203 phylogenetic clusters comprised of 2 to 32 sequences (S1 Fig, S2 Fig and S3 Fig). These clusters were subsequently confirmed by our Bayesian analyses at a P≥0.95. Higher proportions of clustered sequences were found in zip codes where HIV prevalence is highest (r = 0.607; P<0.00001) with southeastern Washington, DC zip codes, being the highest (Fig 3). A network approach (HIV-Trace) grouped 3.6% of the *int* sequences into 4 clusters of 2 sequences and 10.6% of the *PR/RT* sequences into 76 clusters of 2–7 sequences (Fig 4). Hence, this approach identified fewer and smaller clusters compared to the ML-bootstrap approach.

### Phenotypic associations

We found five well-supported (≥70% bootstrap proportion and ≥0.95 posterior probability) *PR/RT* clades of 11 to 32 HIV sequences. None of the clades showed exclusive mapping of a particular risk group (Fig 1). However, our chi-square analyses detected a strong association (P<0.00001) between those five clades and risk (MSM, IDU and HRH) and sex (Table 4), still highly significant after a Bonferroni threshold for multiple tests of P<0.0167. Because MSM and HRH are so highly correlated with sex, the sex significant results may be due to correlation with risk factor. Therefore, we re-ran the test for sex excluding MSM (Table 4) and the evidence for a sex effect dropped dramatically (P = 0.095), suggesting that the sex association was likely due to its correlation with the most common risk categories. The other clinical variables were almost constant across clades (e.g., race/ethnicity) or showed low numbers of cases per cell in our chi-square test (e.g., IDU).

**Table 4:** Chi-square tests of association between phylogenetic clades and demographic variables.

	Risk	Sex	Sex w/o MSM
Clade	Total (% HRH)	Total (% Female)	Total (% Female)
1	11 (18.2%)	11 (0%)	2 (0%)
2	20 (70%)	25 (48%)	16 (68.8%)
3	20 (100%)	22 (81.8%)	21 (85.7%)
4	10 (100%)	11 (63.6%)	10 (70%)
5	12 (0%)	13 (7.7%)	1 (100%)
P-value	<1.00E-08	9.50E-07	0.095

Previous HIV studies in British Columbia (Canada) [39] and Switzerland [40] using intra-patient or inter-patient sampling detected higher numbers of viral sequences falling within clusters (3120–4431, (55–57% of all sequences)) than our study (647 (39.0%) of the *PR/RT* sequences). Similarly, HIV epidemics in those two studies were dominated by 3–5 clusters of 1051–107 sequences and 14–17 clusters of 29–88 sequences. The HIV DC population was grouped into 203 phylogenetic clusters of 2 to 32 *PR/RT* sequences, of which only 5 clusters comprised 11–32 individuals. Ongoing work by our group including intra-patient sequences from multiple patients generated via high-throughput (MiSeq) technology and covering a larger geographic area (DC and neighboring states) will allow us to apply some on the methodologies (e.g., patristic distances) in [39, 40] to identify transmission clusters and consider multiple clinical co-variables simultaneously.

## Discussion

### Molecular surveillance and subtype diversity

The predominant HIV-1 subtype circulating among a subgroup of DC Cohort participants comprised of mainly Non-Hispanic Blacks from 2011–2015 was subtype B, which accounted for 98.7% of the infections for *int* and 93.9% for *PR/RT*. Subtype B is also the predominant subtype in the US and Western Europe. We also detected another 15 subtypes that accounted for the remaining sequences. A study by Kassaye et al. [41] also focused on HIV diversity in DC used *pol* sequences collected from 641 individuals enrolled between 1994–2013 and showed a higher frequency of non-subtype B sequences of HIV (88.5% subtype B and 11.4% non-B HIV-1 subtypes). These differences could be due to demographic and clinical characteristics of the participants in each cohort, changes in the dynamics of HIV in DC over the different time frames of the two studies and/or the fact that the Kassaye et al. study surveyed over a period of time that was double the timeframe of this study. HIV prevalence among ethnic minorities is very high in DC, much less among the heterosexual white population, indicating that the HIV epidemic is concentrated in key populations defined by sexual orientation and ethnicity. Yet, nearly all patients harbored subtype B viruses, which suggests that the local epidemic amongst ethnic minorities was seeded in the US and is at present not primarily driven by importation from sub-Saharan Africa or other parts of the world where non-B subtypes circulate. The finding of a small proportion of patients with non-subtype B virus, nonetheless, highlights the cultural diversity of DC and the immigration of persons from other areas of the world. Higher diversity of non-B HIV-1 subtypes has been reported in other immigrant-rich North American cohorts on the East Coast such as Rhode Island (8.3%) [42], Maryland (12.9%) [6], and New York City (43.4%) [43]. Similarly, the prevalence of non-B infections has increased from 5.9% in 2006 to 8.5% in 2013 in 7 US states (Colorado, Connecticut, Michigan, New York, South Carolina, Texas, and Washington) [44]. Lower rates, however, of non-B subtypes have been reported for other Southeastern US states like North Carolina [45], which is consistent with the national average prevalence of non-B subtypes (3.27%) estimated in 2011 [46].

Our estimates of *int* and *PR/RT* genetic diversity in subtype B viruses from DC were relatively high (e.g., θ = 0.08–0.09, for all main subtypes) compared to other US subtype B strains available in Los Alamos HIV database for *int* (θ = 0.075) and *PR/RT* (θ = 0.067). This suggests that the HIV epidemic in DC is likely to be mature and that extensive exchange between risk groups has been ongoing for years, which is also supported by our phylodynamic results as discussed below. High HIV genetic and subtype diversity is of concern as it complicates vaccine development by increasing the chances for the evolution of vaccine resistance or the failure for specific epitopes to work against broad diversity [47].

Our population estimators of genetic diversity, recombination, and selection showed differences between patients grouped by risk factor for some estimators. This may suggest differences in transmission and HIV-1 dynamics among risk groups. HIV Subtype B in perinatally infected participants showed higher levels of diversity compared to other risk groups likely due to the uniqueness of mother-infant transmission in perinatal infections (i.e., potentially high HIV effective population size) and exposure to many different ARV regimens.

Our phylodynamic analyses of the US born subtype B sequences showed that the relative genetic diversity of the DC viral population has not significantly decreased over the last 25 years. This is consistent with previous phylodynamic analyses of US subtype B sequences collected between 1981 and 2006 [47]. More importantly, the epidemic persists among certain groups like MSM, where infection rates are still very high and continue to be a source of continued transmission [1, 3, 47]. Therefore, department of health prevention efforts should continue to focus on high-risk groups as well as the general population [1, 48].

### Phylogenetic structure of HIV-1 in Washington DC

Our phylogenetic analyses of subtype B sequences did not reveal clear evidence that HIV-1 populations in DC are structured by any of the epidemiological and clinical factors studied. These results agree with previous subtype B star-like phylogenies reported for DC [41]. Geographically broader phylogenetic studies across the US also showed lack of phylogenetic structuring based on transmission type, sociodemographic factors, and geographic location [47]. Keele et al. [49], for example, showed that viral *env* genes evolving from individual transmitted or founder HIV-1 subtype B viruses generally exhibited a star-like phylogeny, such as the one observed in North American viruses [47]. Given the maturity of the HIV-1 epidemic in DC and the fact that the virus is thought to mutate at a rate of 1% per year [50, 51], the possibility exists that different clades could have emerged in different wards or high-risk groups in the city. Indeed, phylogenetic structuring based on these factors has been observed before between subtypes in, for example, Africa [52] and Asia [53], and within subtypes in, for example, Vietnam [54] and China [55]. But contrary to what happened in those HIV/AIDS epidemics, our star-like gene genealogies of HIV-1 in Washington DC suggest the DC epidemic expanded uniformly (i.e., no phylogenetic structure) across the metropolitan area and across the epidemiological risk types [56, 57].

### Transmission networks

The extent to which transmission of HIV-1 is clustered is not clear. Some studies [58–69] report high clustering (24 to 65%) levels, while others [18, 47, 67] show much lower values (7 to 17%) for the same subtypes and transmission routes. Our comprehensive phylogenetic analyses of HIV-1 from DC show moderate proportions of subtype B infections (13.5% to 39.0% depending on the gene) falling into clusters, confirming that transmission chains play a role in HIV-1 transmission and spread of HIV in DC [41]. Previous studies of HIV-1 clustering in DC and North America reported values of ~17% [41, 47], but a recent study of 86 patients in Chicago showed levels of 36% [69]. Moreover, differences in clustering have also been observed between subtypes, transmission routes, mental health and geographic regions [47, 62, 63, 69]. Our phenotypic association testing (chi-square) showed significant (P<0.00001) association between *PR/RT* clusters and risk factor (transmission group), suggesting that MSM and HRH may comprise largely independent transmission networks in the DC area, as seen in other US states [44, 70].

### Evolution of drug resistance

We detected a high prevalence of Drug Resistance Mutations (DRM) for both subtype B *int* (17.1%) and *PR/RT* (39.1%) with similar prevalence across risk groups except for among heterosexuals when looking at *int*, (2.6%) and perinatally infected participants when looking at *PR/RT* (68.4%). High rates of DRM were also detected in subtype BD (34.5%) and C (25.0%) for *PR/RT*. *PR/RT* prevalence rates reported in this study are higher than those previously reported for the DC area in 1994–2013 using *pol* sequences [41]. DRM rates higher than 50% are frequently observed in large sequence databases in Europe (e.g., UK, http://www.hivrdb.org.uk and Switzerland, www.shcs.ch) when similar patient groups are considered (i.e., ART experienced). This finding may also reflect patient selection. Future analyses should determine if DRMs of current treatment regimens cluster, and if there is evidence for increasing prevalence of DRMs based on current treatment regimens in the DC area. Similarly, studies including only naïve patients are needed to determine to what extent these DRM are actually being transmitted in the DC area.

Finally, we also detected 8 *int* and 12 *PR/RT* amino acids under adaptive selection. These correspond to sites evolving in the DC HIV-1 subtype B population that have not been fixed in HIV-1 as those conferring drug resistance. These amino acid sites involved positions that, in the future, may confer resistance to antiretrovirals if they become fixed in the population and could be helpful in informing ARV regimen choices.

### Conclusions

Routinely collected commercial sequences are useful for examining transmission dynamics and can provide an historical context for the HIV epidemic. Our ability to combine them with surveillance, clinical, and demographic indicators enhances their utility in understanding transmission patterns and geospatial distribution. Given the large number of sequences analyzed, the data presented in this study inform our understanding of the molecular epidemiology of HIV infection in Washington DC. Moreover, they help lay the foundation for future work in which molecular and epidemiologic data could be used synergistically to target public health interventions to interrupt HIV transmission networks.

## Supporting information

S1 Table Amino acid mutations counted as drug resistant mutations for each main subtype and gene region analyzed in this study. (XLSX)

S1 Fig Maximum likelihood phylogenetic tree of Washington DC HIV-1 *int* isolates. Clades supported by bootstrap proportions ≥70% are indicated with an asterisk. These clades were also supported by Bayesian posterior probabilities ≥0.95.
(PDF)

S2 Fig Maximum likelihood phylogenetic tree of Washington DC HIV-1 *PR/RT* isolates. (PDF)

S3 Fig Maximum likelihood consensus phylogenetic tree of Washington DC HIV-1 *PR/RT* isolates showing clades supported by bootstrap proportions ≥70%. These clades were also supported by Bayesian posterior probabilities ≥0.95.
(PDF)
