# Supplementary figures and images for: Characterization of HIV diversity, phylodynamics and drug resistance in Washington, DC
Source: PLoS One. 2017 Sep 29;12(9):e0185644. doi: 10.1371/journal.pone.0185644 (PMC5621693; doi:10.1371/journal.pone.0185644)

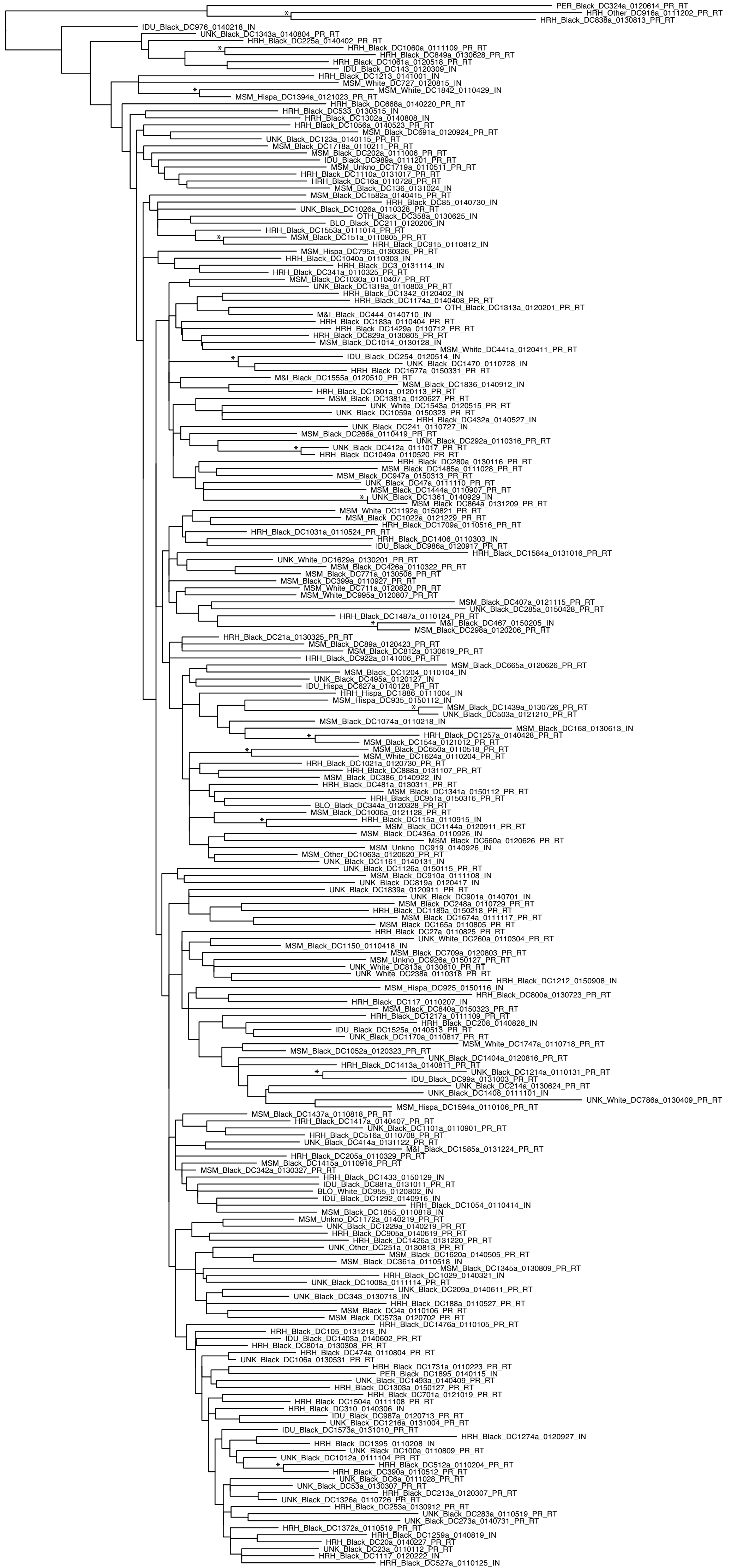

0.04

Supplement: S1 Fig — Clades supported by bootstrap proportions ≥70% are indicated with an asterisk. These clades were also supported by Bayesian posterior probabilities ≥0.95. (PDF) [file pone.0185644.s002.pdf]
