# Supplementary material for: Characterization of HIV diversity, phylodynamics and drug resistance in Washington, DC
Source: PLoS One. 2017 Sep 29;12(9):e0185644. doi: 10.1371/journal.pone.0185644 (PMC5621693; doi:10.1371/journal.pone.0185644)
Supplement: S3 Fig — These clades were also supported by Bayesian posterior probabilities ≥0.95. (PDF) [file pone.0185644.s004.pdf]

Majority-rule consensus tree

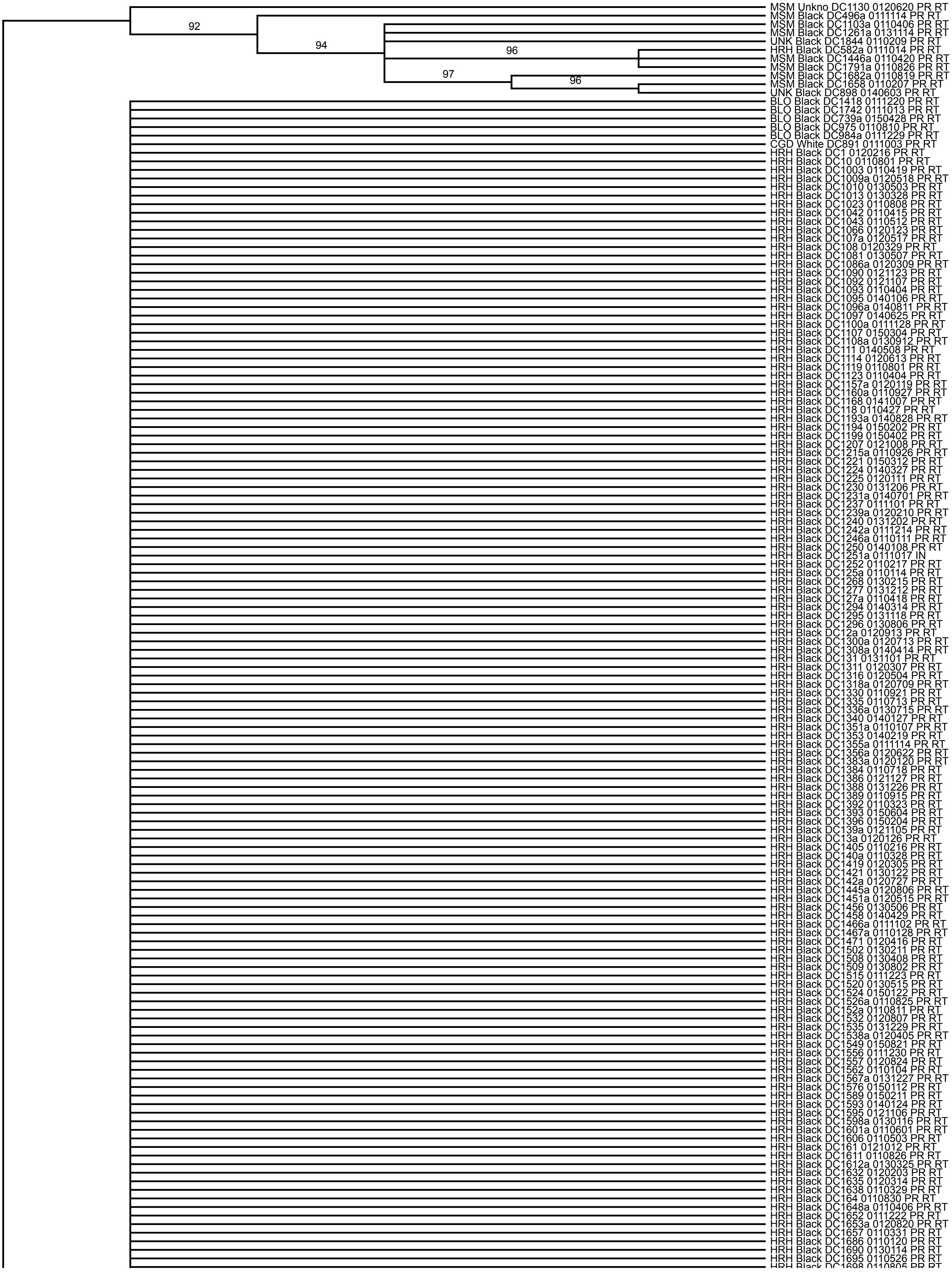

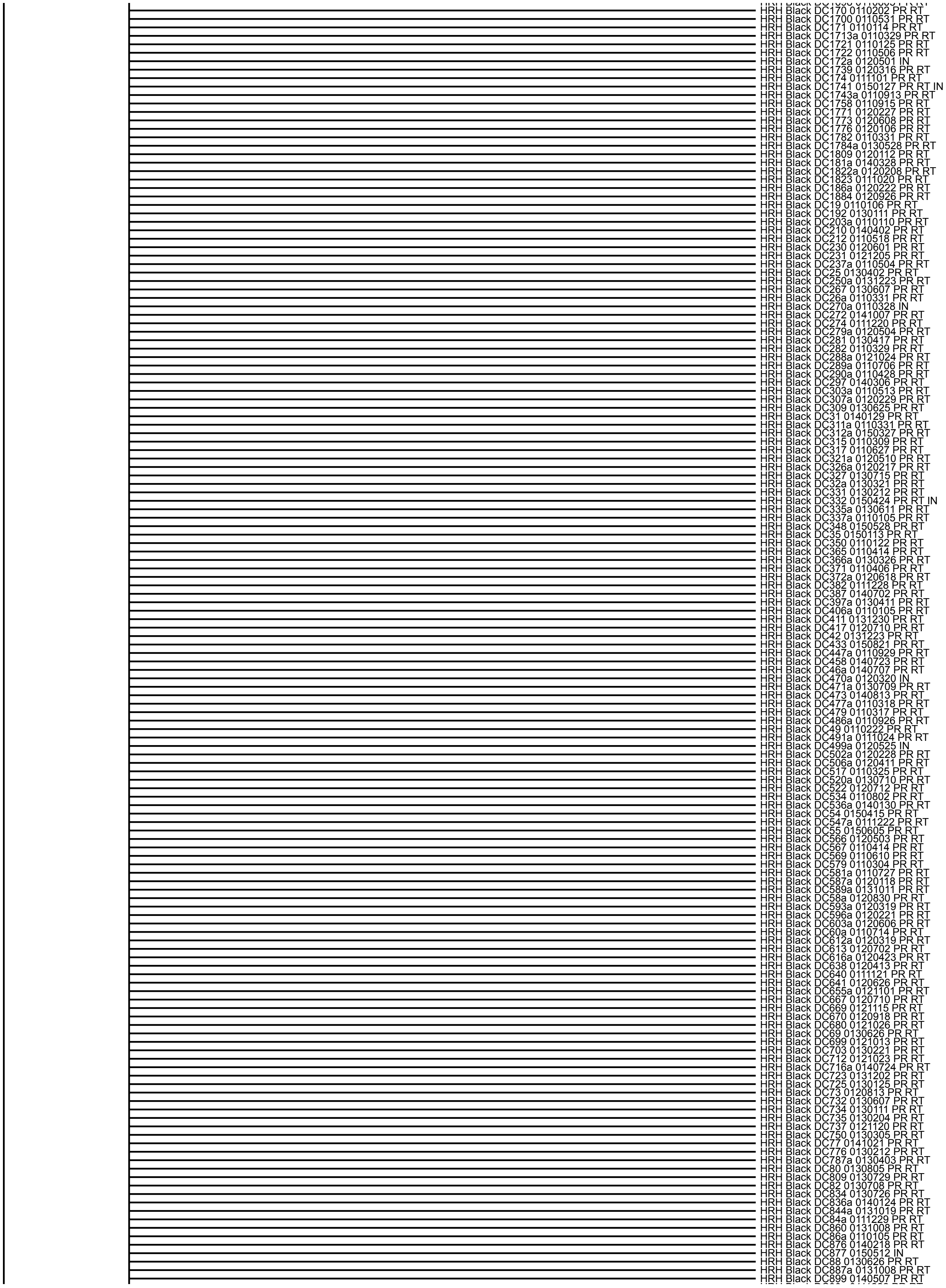

HRH Black DC170 0110202 PR RT
HRH Black DC1700 0110531 PR RT
HRH Black DC171 0110114 PR RT
HRH Black DC1713a 0110329 PR RT
HRH Black DC1721 0110125 PR RT
HRH Black DC1722 0110506 PR RT
HRH Black DC172a 0120501 IN
HRH Black DC1739 0120316 PR RT
HRH Black DC174 0111101 PR RT
HRH Black DC1741 0150127 PR RT IN
HRH Black DC1743a 0110913 PR RT
HRH Black DC1758 0110915 PR RT
HRH Black DC1771 0120227 PR RT
HRH Black DC1773 0120608 PR RT
HRH Black DC1776 0120106 PR RT
HRH Black DC1782 0110331 PR RT
HRH Black DC1784a 0130528 PR RT
HRH Black DC1809 0120112 PR RT
HRH Black DC181a 0140328 PR RT
HRH Black DC1822a 0120208 PR RT
HRH Black DC1823 0111020 PR RT
HRH Black DC186a 0120222 PR RT
HRH Black DC1884 0120926 PR RT
HRH Black DC19 0110106 PR RT
HRH Black DC192 0130111 PR RT
HRH Black DC203a 0110110 PR RT
HRH Black DC210 0140402 PR RT
HRH Black DC212 0110518 PR RT
HRH Black DC230 0120601 PR RT
HRH Black DC231 0121205 PR RT
HRH Black DC237a 0110504 PR RT
HRH Black DC25 0130402 PR RT
HRH Black DC250a 0131223 PR RT
HRH Black DC267 0130607 PR RT
HRH Black DC26a 0110331 PR RT
HRH Black DC270a 0110328 IN
HRH Black DC272 0141007 PR RT
HRH Black DC274 0111220 PR RT
HRH Black DC279a 0120504 PR RT
HRH Black DC281 0130417 PR RT
HRH Black DC282 0110329 PR RT
HRH Black DC288a 0121024 PR RT
HRH Black DC289a 0110706 PR RT
HRH Black DC290a 0110428 PR RT
HRH Black DC297 0140306 PR RT
HRH Black DC303a 0110513 PR RT
HRH Black DC307a 0120229 PR RT
HRH Black DC309 0130625 PR RT
HRH Black DC31 0140129 PR RT
HRH Black DC311a 0110331 PR RT
HRH Black DC312a 0150327 PR RT
HRH Black DC315 0110309 PR RT
HRH Black DC317 0110627 PR RT
HRH Black DC321a 0120510 PR RT
HRH Black DC326a 0120217 PR RT
HRH Black DC327 0130715 PR RT
HRH Black DC32a 0130321 PR RT
HRH Black DC331 0130212 PR RT
HRH Black DC332 0150424 PR RT IN
HRH Black DC335a 0130611 PR RT
HRH Black DC337a 0110105 PR RT
HRH Black DC348 0150528 PR RT
HRH Black DC35 0150113 PR RT
HRH Black DC350 0110122 PR RT
HRH Black DC365 0110414 PR RT
HRH Black DC366a 0130326 PR RT
HRH Black DC371 0110406 PR RT
HRH Black DC372a 0120618 PR RT
HRH Black DC382 0111228 PR RT
HRH Black DC387 0140702 PR RT
HRH Black DC397a 0130411 PR RT
HRH Black DC406a 0110105 PR RT
HRH Black DC411 0131230 PR RT
HRH Black DC417 0120710 PR RT
HRH Black DC42 0131223 PR RT
HRH Black DC433 0150821 PR RT
HRH Black DC447a 0110929 PR RT
HRH Black DC458 0140723 PR RT
HRH Black DC46a 0140707 PR RT
HRH Black DC470a 0120320 IN
HRH Black DC471a 0130709 PR RT
HRH Black DC473 0140813 PR RT
HRH Black DC477a 0110318 PR RT
HRH Black DC479 0110317 PR RT
HRH Black DC486a 0110926 PR RT
HRH Black DC49 0110222 PR RT
HRH Black DC491a 0111024 PR RT
HRH Black DC499a 0120525 IN
HRH Black DC502a 0120228 PR RT
HRH Black DC506a 0120411 PR RT
HRH Black DC517 0110325 PR RT
HRH Black DC520a 0130710 PR RT
HRH Black DC522 0120712 PR RT
HRH Black DC534 0110802 PR RT
HRH Black DC536a 0140130 PR RT
HRH Black DC54 0150415 PR RT
HRH Black DC547a 0111222 PR RT
HRH Black DC55 0150605 PR RT
HRH Black DC566 0120503 PR RT
HRH Black DC567 0110414 PR RT
HRH Black DC569 0110610 PR RT
HRH Black DC579 0110304 PR RT
HRH Black DC581a 0110727 PR RT
HRH Black DC587a 0120118 PR RT
HRH Black DC589a 0131011 PR RT
HRH Black DC58a 0120830 PR RT
HRH Black DC593a 0120319 PR RT
HRH Black DC596a 0120221 PR RT
HRH Black DC603a 0120606 PR RT
HRH Black DC60a 0110714 PR RT
HRH Black DC612a 0120319 PR RT
HRH Black DC613 0120702 PR RT
HRH Black DC616a 0120423 PR RT
HRH Black DC638 0120413 PR RT
HRH Black DC640 0111121 PR RT
HRH Black DC641 0120626 PR RT
HRH Black DC655a 0121101 PR RT
HRH Black DC667 0120710 PR RT
HRH Black DC669 0121115 PR RT
HRH Black DC670 0120918 PR RT
HRH Black DC680 0121026 PR RT
HRH Black DC69 0130626 PR RT
HRH Black DC699 0121013 PR RT
HRH Black DC703 0130221 PR RT
HRH Black DC712 0121023 PR RT
HRH Black DC716a 0140724 PR RT
HRH Black DC723 0131202 PR RT
HRH Black DC725 0130125 PR RT
HRH Black DC73 0120813 PR RT
HRH Black DC732 0130607 PR RT
HRH Black DC734 0130111 PR RT
HRH Black DC735 0130204 PR RT
HRH Black DC737 0121120 PR RT
HRH Black DC750 0130305 PR RT
HRH Black DC77 0141021 PR RT
HRH Black DC776 0130212 PR RT
HRH Black DC787a 0130403 PR RT
HRH Black DC80 0130805 PR RT
HRH Black DC809 0130729 PR RT
HRH Black DC82 0130708 PR RT
HRH Black DC834 0130726 PR RT
HRH Black DC836a 0140124 PR RT
HRH Black DC844a 0131019 PR RT
HRH Black DC84a 0111229 PR RT
HRH Black DC860 0131008 PR RT
HRH Black DC86a 0110105 PR RT
HRH Black DC876 0140218 PR RT
HRH Black DC877 0150512 IN
HRH Black DC88 0130626 PR RT
HRH Black DC887a 0131008 PR RT
HRH Black DC899 0140507 PR RT

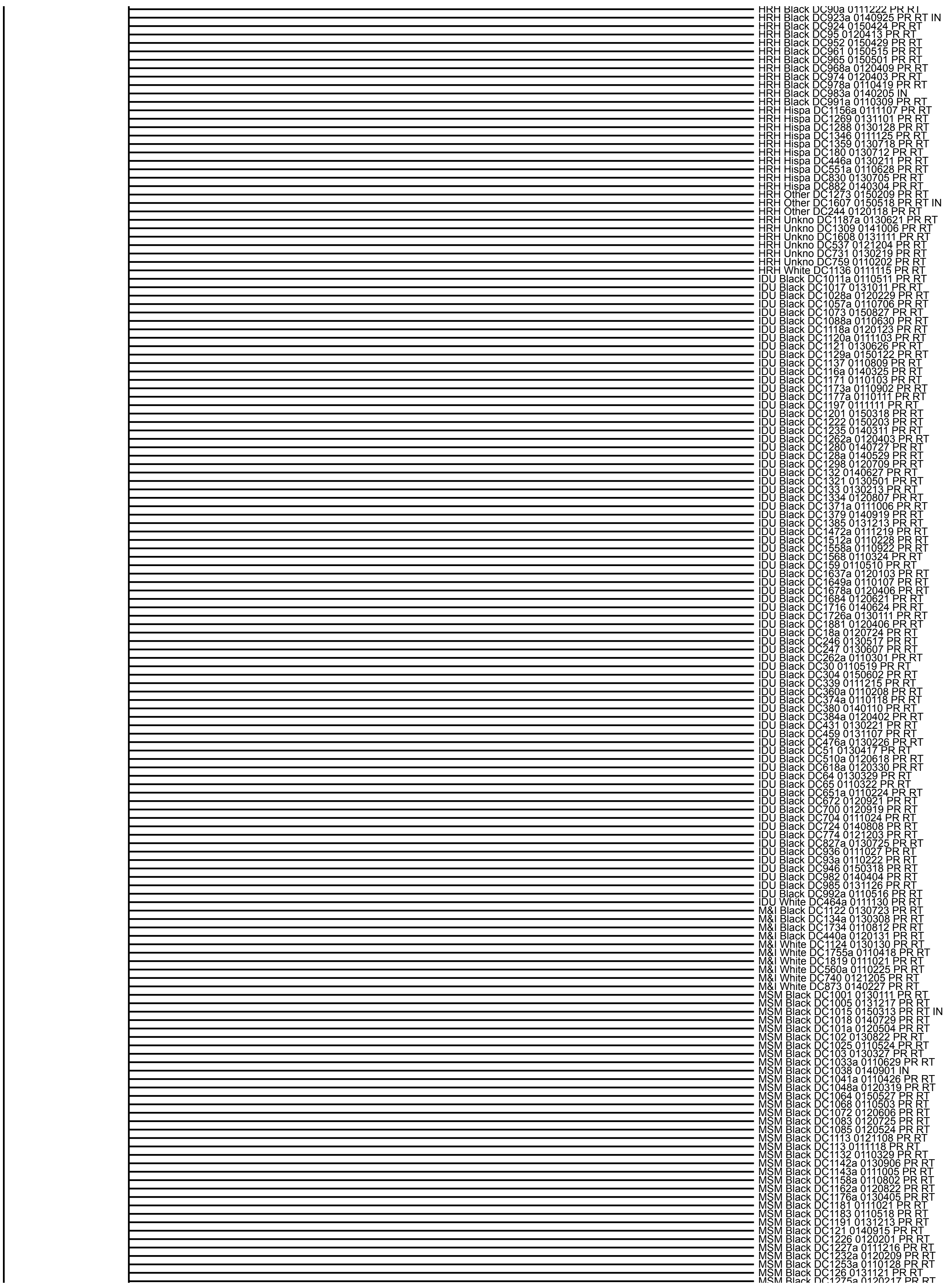

HRH Black DC90a 0111222 PR RT
HRH Black DC923a 0140925 PR RT IN
HRH Black DC924 0150424 PR RT
HRH Black DC95 0120413 PR RT
HRH Black DC952 0150429 PR RT
HRH Black DC961 0150515 PR RT
HRH Black DC965 0150501 PR RT
HRH Black DC968a 0120409 PR RT
HRH Black DC974 0120403 PR RT
HRH Black DC978a 0110419 PR RT
HRH Black DC983a 0140205 IN
HRH Black DC991a 0110309 PR RT
HRH Hispa DC1156a 0111107 PR RT
HRH Hispa DC1269 0131101 PR RT
HRH Hispa DC1288 0130128 PR RT
HRH Hispa DC1346 0111125 PR RT
HRH Hispa DC1359 0130718 PR RT
HRH Hispa DC180 0130712 PR RT
HRH Hispa DC446a 0130211 PR RT
HRH Hispa DC551a 0110628 PR RT
HRH Hispa DC830 0130705 PR RT
HRH Hispa DC882 0140304 PR RT
HRH Other DC1273 0150209 PR RT
HRH Other DC1607 0150518 PR RT IN
HRH Other DC244 0120118 PR RT
HRH Unkno DC1187a 0130621 PR RT
HRH Unkno DC1309 0141006 PR RT
HRH Unkno DC1608 0131111 PR RT
HRH Unkno DC537 0121204 PR RT
HRH Unkno DC731 0130219 PR RT
HRH Unkno DC759 0110202 PR RT
HRH White DC1136 0111115 PR RT
IDU Black DC1011a 0110511 PR RT
IDU Black DC1017 0131011 PR RT
IDU Black DC1028a 0120229 PR RT
IDU Black DC1057a 0110706 PR RT
IDU Black DC1073 0150827 PR RT
IDU Black DC1088a 0110630 PR RT
IDU Black DC1118a 0120123 PR RT
IDU Black DC1120a 0111103 PR RT
IDU Black DC1121 0130626 PR RT
IDU Black DC1129a 0150122 PR RT
IDU Black DC1137 0110809 PR RT
IDU Black DC116a 0140325 PR RT
IDU Black DC1171 0110103 PR RT
IDU Black DC1173a 0110902 PR RT
IDU Black DC1177a 0110111 PR RT
IDU Black DC1197 0111111 PR RT
IDU Black DC1201 0150318 PR RT
IDU Black DC1222 0150203 PR RT
IDU Black DC1235 0140311 PR RT
IDU Black DC1262a 0120403 PR RT
IDU Black DC1280 0140727 PR RT
IDU Black DC128a 0140529 PR RT
IDU Black DC1298 0120709 PR RT
IDU Black DC132 0140627 PR RT
IDU Black DC1321 0130501 PR RT
IDU Black DC133 0130213 PR RT
IDU Black DC1334 0120807 PR RT
IDU Black DC1371a 0111006 PR RT
IDU Black DC1379 0140919 PR RT
IDU Black DC1385 0131213 PR RT
IDU Black DC1472a 0111219 PR RT
IDU Black DC1512a 0110228 PR RT
IDU Black DC1558a 0110922 PR RT
IDU Black DC1568 0110324 PR RT
IDU Black DC159 0110510 PR RT
IDU Black DC1637a 0120103 PR RT
IDU Black DC1649a 0110107 PR RT
IDU Black DC1678a 0120406 PR RT
IDU Black DC1684 0120621 PR RT
IDU Black DC1716 0140624 PR RT
IDU Black DC1726a 0130111 PR RT
IDU Black DC1881 0120406 PR RT
IDU Black DC18a 0120724 PR RT
IDU Black DC246 0130517 PR RT
IDU Black DC247 0130607 PR RT
IDU Black DC262a 0110301 PR RT
IDU Black DC30 0110519 PR RT
IDU Black DC304 0150602 PR RT
IDU Black DC339 0111215 PR RT
IDU Black DC360a 0110208 PR RT
IDU Black DC374a 0110118 PR RT
IDU Black DC380 0140110 PR RT
IDU Black DC384a 0120402 PR RT
IDU Black DC431 0130221 PR RT
IDU Black DC459 0131107 PR RT
IDU Black DC476a 0130226 PR RT
IDU Black DC51 0130417 PR RT
IDU Black DC510a 0120618 PR RT
IDU Black DC618a 0120330 PR RT
IDU Black DC64 0130329 PR RT
IDU Black DC65 0110322 PR RT
IDU Black DC651a 0110224 PR RT
IDU Black DC672 0120921 PR RT
IDU Black DC700 0120919 PR RT
IDU Black DC704 0111024 PR RT
IDU Black DC724 0140808 PR RT
IDU Black DC774 0121203 PR RT
IDU Black DC827a 0130725 PR RT
IDU Black DC936 0111027 PR RT
IDU Black DC93a 0110222 PR RT
IDU Black DC946 0150318 PR RT
IDU Black DC982 0140404 PR RT
IDU Black DC985 0131126 PR RT
IDU Black DC992a 0110516 PR RT
IDU White DC464a 0111130 PR RT
M&I Black DC1122 0130723 PR RT
M&I Black DC134a 0130308 PR RT
M&I Black DC1734 0110812 PR RT
M&I Black DC440a 0120131 PR RT
M&I White DC1124 0130130 PR RT
M&I White DC1755a 0110418 PR RT
M&I White DC1819 0111021 PR RT
M&I White DC560a 0110225 PR RT
M&I White DC740 0121205 PR RT
M&I White DC873 0140227 PR RT
MSM Black DC1001 0130111 PR RT
MSM Black DC1005 0131217 PR RT
MSM Black DC1015 0150313 PR RT IN
MSM Black DC1018 0140729 PR RT
MSM Black DC101a 0120504 PR RT
MSM Black DC102 0130822 PR RT
MSM Black DC1025 0110524 PR RT
MSM Black DC103 0130327 PR RT
MSM Black DC1033a 0110629 PR RT
MSM Black DC1038 0140901 IN
MSM Black DC1041a 0110426 PR RT
MSM Black DC1048a 0120319 PR RT
MSM Black DC1064 0150527 PR RT
MSM Black DC1068 0110503 PR RT
MSM Black DC1072 0120606 PR RT
MSM Black DC1083 0120725 PR RT
MSM Black DC1085 0120524 PR RT
MSM Black DC1113 0121108 PR RT
MSM Black DC113 0111118 PR RT
MSM Black DC1132 0110329 PR RT
MSM Black DC1142a 0130906 PR RT
MSM Black DC1143a 0111005 PR RT
MSM Black DC1158a 0110802 PR RT
MSM Black DC1162a 0120822 PR RT
MSM Black DC1176a 0130405 PR RT
MSM Black DC1181 0111021 PR RT
MSM Black DC1183 0110518 PR RT
MSM Black DC1191 0131213 PR RT
MSM Black DC121 0140915 PR RT
MSM Black DC1226 0120201 PR RT
MSM Black DC1227a 0111216 PR RT
MSM Black DC1232a 0120209 PR RT
MSM Black DC1253a 0110128 PR RT
MSM Black DC126 0131121 PR RT
MSM Black DC1275a 0120217 PR RT

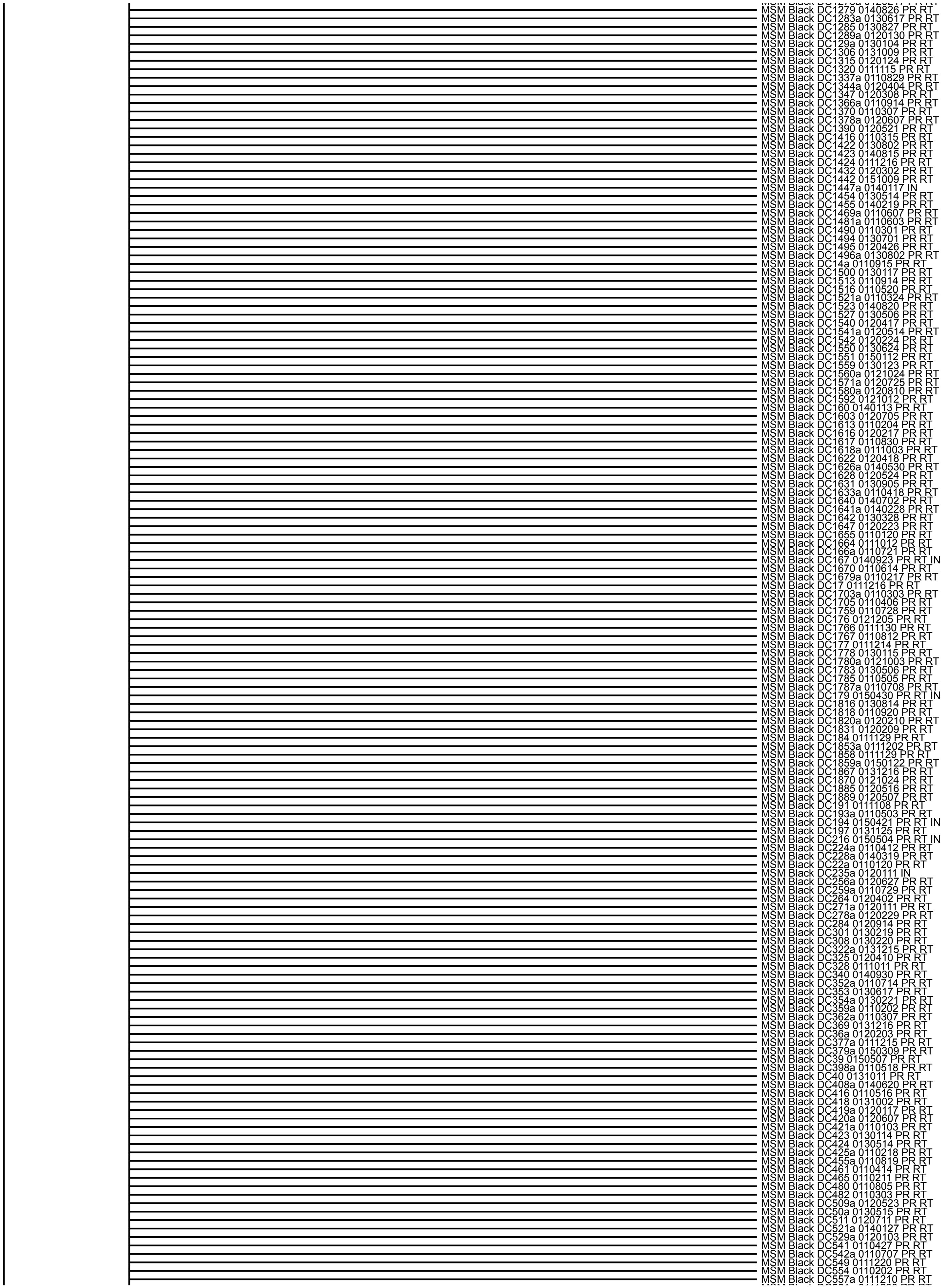
MSM Black DC1279 0140826 PR RT
MSM Black DC1283a 0130617 PR RT
MSM Black DC1285 0130827 PR RT
MSM Black DC1289a 0120130 PR RT
MSM Black DC129a 0130104 PR RT
MSM Black DC1306 0131009 PR RT
MSM Black DC1315 0120124 PR RT
MSM Black DC1320 0111115 PR RT
MSM Black DC1337a 0110829 PR RT
MSM Black DC1344a 0120404 PR RT
MSM Black DC1347 0120308 PR RT
MSM Black DC1366a 0110914 PR RT
MSM Black DC1370 0110307 PR RT
MSM Black DC1378a 0120607 PR RT
MSM Black DC1390 0120521 PR RT
MSM Black DC1416 0110315 PR RT
MSM Black DC1422 0130802 PR RT
MSM Black DC1423 0140815 PR RT
MSM Black DC1424 0111216 PR RT
MSM Black DC1432 0120302 PR RT
MSM Black DC1442 0151009 PR RT
MSM Black DC1447a 0140117 IN
MSM Black DC1454 0130514 PR RT
MSM Black DC1455 0140219 PR RT
MSM Black DC1469a 0110607 PR RT
MSM Black DC1481a 0110603 PR RT
MSM Black DC1490 0110301 PR RT
MSM Black DC1494 0130701 PR RT
MSM Black DC1495 0120426 PR RT
MSM Black DC1496a 0130802 PR RT
MSM Black DC14a 0110915 PR RT
MSM Black DC1500 0130117 PR RT
MSM Black DC1513 0110914 PR RT
MSM Black DC1516 0110520 PR RT
MSM Black DC1521a 0110324 PR RT
MSM Black DC1523 0140820 PR RT
MSM Black DC1527 0130506 PR RT
MSM Black DC1540 0120417 PR RT
MSM Black DC1541a 0120514 PR RT
MSM Black DC1542 0120224 PR RT
MSM Black DC1550 0130624 PR RT
MSM Black DC1551 0150112 PR RT
MSM Black DC1559 0130123 PR RT
MSM Black DC1560a 0121024 PR RT
MSM Black DC1571a 0120725 PR RT
MSM Black DC1580a 0120810 PR RT
MSM Black DC1592 0121012 PR RT
MSM Black DC160 0140113 PR RT
MSM Black DC1603 0120705 PR RT
MSM Black DC1613 0110204 PR RT
MSM Black DC1616 0120217 PR RT
MSM Black DC1617 0110830 PR RT
MSM Black DC1618a 0111003 PR RT
MSM Black DC1622 0120418 PR RT
MSM Black DC1626a 0140530 PR RT
MSM Black DC1628 0120524 PR RT
MSM Black DC1631 0130905 PR RT
MSM Black DC1633a 0110418 PR RT
MSM Black DC1640 0140702 PR RT
MSM Black DC1641a 0140228 PR RT
MSM Black DC1642 0130328 PR RT
MSM Black DC1647 0120223 PR RT
MSM Black DC1655 0110120 PR RT
MSM Black DC1664 0111012 PR RT
MSM Black DC166a 0110721 PR RT
MSM Black DC167 0140923 PR RT IN
MSM Black DC1670 0110614 PR RT
MSM Black DC1679a 0110217 PR RT
MSM Black DC17 0111216 PR RT
MSM Black DC1703a 0110303 PR RT
MSM Black DC1705 0110406 PR RT
MSM Black DC1759 0110728 PR RT
MSM Black DC176 0121205 PR RT
MSM Black DC1766 0111130 PR RT
MSM Black DC1767 0110812 PR RT
MSM Black DC177 0111214 PR RT
MSM Black DC1778 0130115 PR RT
MSM Black DC1780a 0121003 PR RT
MSM Black DC1783 0130506 PR RT
MSM Black DC1785 0110505 PR RT
MSM Black DC1787a 0110708 PR RT
MSM Black DC179 0150430 PR RT IN
MSM Black DC1816 0130814 PR RT
MSM Black DC1818 0110920 PR RT
MSM Black DC1820a 0120210 PR RT
MSM Black DC1831 0120209 PR RT
MSM Black DC184 0111129 PR RT
MSM Black DC1853a 0111202 PR RT
MSM Black DC1858 0111129 PR RT
MSM Black DC1859a 0150122 PR RT
MSM Black DC1867 0131216 PR RT
MSM Black DC1870 0121024 PR RT
MSM Black DC1885 0120516 PR RT
MSM Black DC1889 0120507 PR RT
MSM Black DC191 0111108 PR RT
MSM Black DC193a 0110503 PR RT
MSM Black DC194 0150421 PR RT IN
MSM Black DC197 0131125 PR RT
MSM Black DC216 0150504 PR RT IN
MSM Black DC224a 0110412 PR RT
MSM Black DC228a 0140319 PR RT
MSM Black DC22a 0110120 PR RT
MSM Black DC235a 0120111 IN
MSM Black DC256a 0120627 PR RT
MSM Black DC259a 0110729 PR RT
MSM Black DC264 0120402 PR RT
MSM Black DC271a 0120111 PR RT
MSM Black DC278a 0120229 PR RT
MSM Black DC284 0120914 PR RT
MSM Black DC301 0130219 PR RT
MSM Black DC308 0130220 PR RT
MSM Black DC322a 0131215 PR RT
MSM Black DC325 0120410 PR RT
MSM Black DC328 0111011 PR RT
MSM Black DC340 0140930 PR RT
MSM Black DC352a 0110714 PR RT
MSM Black DC353 0130617 PR RT
MSM Black DC354a 0130221 PR RT
MSM Black DC359a 0110202 PR RT
MSM Black DC362a 0110307 PR RT
MSM Black DC369 0131216 PR RT
MSM Black DC36a 0120203 PR RT
MSM Black DC377a 0111215 PR RT
MSM Black DC379a 0150309 PR RT
MSM Black DC39 0150507 PR RT
MSM Black DC398a 0110518 PR RT
MSM Black DC40 0131011 PR RT
MSM Black DC408a 0140620 PR RT
MSM Black DC416 0110516 PR RT
MSM Black DC418 0131002 PR RT
MSM Black DC419a 0120117 PR RT
MSM Black DC420a 0120607 PR RT
MSM Black DC421a 0110103 PR RT
MSM Black DC423 0130114 PR RT
MSM Black DC424 0130514 PR RT
MSM Black DC425a 0110218 PR RT
MSM Black DC455a 0110819 PR RT
MSM Black DC461 0110414 PR RT
MSM Black DC465 0110211 PR RT
MSM Black DC480 0110805 PR RT
MSM Black DC482 0110303 PR RT
MSM Black DC509a 0120523 PR RT
MSM Black DC50a 0130515 PR RT
MSM Black DC511 0120711 PR RT
MSM Black DC521a 0140127 PR RT
MSM Black DC529a 0120103 PR RT
MSM Black DC541 0110427 PR RT
MSM Black DC542a 0110707 PR RT
MSM Black DC549 0111220 PR RT
MSM Black DC554 0110202 PR RT
MSM Black DC557a 0111210 PR RT

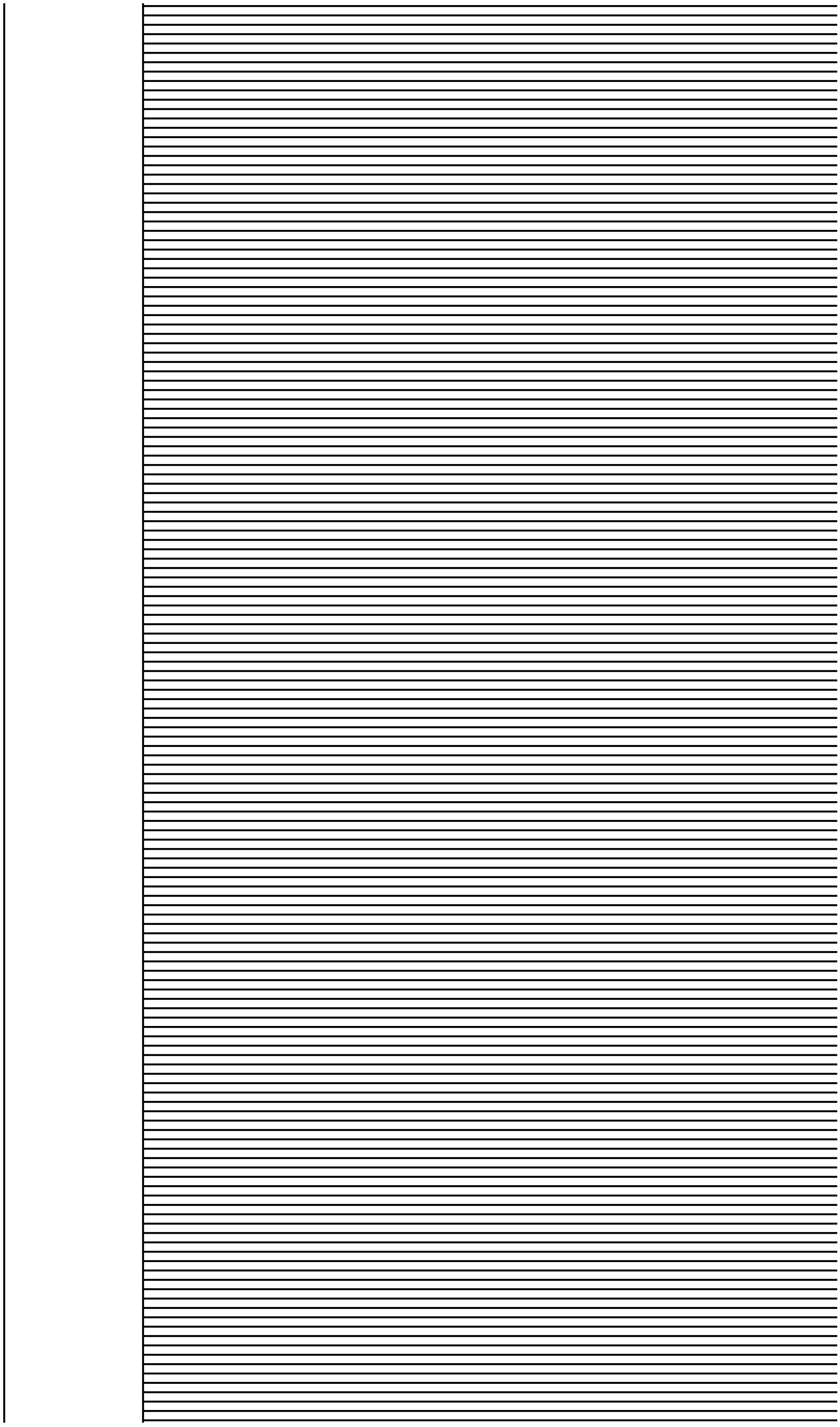
MSM Black DC561a 0111230 PR RT
MSM Black DC563 0110511 PR RT
MSM Black DC572 0121112 PR RT
MSM Black DC577 0110708 PR RT
MSM Black DC578a 0110105 PR RT
MSM Black DC584 0120112 PR RT
MSM Black DC600a 0130924 PR RT
MSM Black DC604 0121102 PR RT
MSM Black DC605 0120703 PR RT
MSM Black DC606 0120209 PR RT
MSM Black DC609 0120402 PR RT
MSM Black DC61 0130910 PR RT
MSM Black DC614a 0120820 PR RT
MSM Black DC62a 0120321 PR RT
MSM Black DC63 0110620 PR RT
MSM Black DC632 0120301 PR RT
MSM Black DC636 0110329 PR RT
MSM Black DC642a 0111025 PR RT
MSM Black DC643 0130709 PR RT
MSM Black DC657a 0120619 PR RT
MSM Black DC67 0110519 PR RT
MSM Black DC676 0120727 PR RT
MSM Black DC677 0120725 PR RT
MSM Black DC678 0121211 PR RT
MSM Black DC679 0120813 PR RT
MSM Black DC68 0130830 PR RT
MSM Black DC683 0120224 PR RT
MSM Black DC688 0131216 PR RT
MSM Black DC693 0120816 PR RT
MSM Black DC697 0131120 PR RT
MSM Black DC70 0111121 PR RT
MSM Black DC706 0130711 PR RT
MSM Black DC71 0110920 PR RT
MSM Black DC718 0130108 PR RT
MSM Black DC720 0121017 PR RT
MSM Black DC738 0121114 PR RT
MSM Black DC741 0130102 PR RT
MSM Black DC745 0121126 PR RT
MSM Black DC747a 0110608 PR RT
MSM Black DC748 0121204 PR RT
MSM Black DC751 0130125 PR RT
MSM Black DC754 0150507 PR RT
MSM Black DC755 0131213 PR RT
MSM Black DC768 0120515 PR RT
MSM Black DC769a 0130123 PR RT
MSM Black DC785 0110914 PR RT
MSM Black DC789a 0130125 PR RT
MSM Black DC791 0130308 PR RT
MSM Black DC793 0130419 PR RT
MSM Black DC797 0130524 PR RT
MSM Black DC798a 0110211 PR RT
MSM Black DC811 0130703 PR RT
MSM Black DC815 0130611 PR RT
MSM Black DC821 0130903 PR RT
MSM Black DC823 0130621 PR RT
MSM Black DC824a 0130624 PR RT
MSM Black DC831 0121207 PR RT
MSM Black DC832 0130709 PR RT
MSM Black DC843 0130905 PR RT
MSM Black DC847 0150805 PR RT
MSM Black DC850 0111024 PR RT
MSM Black DC851 0110729 PR RT
MSM Black DC852 0120416 PR RT
MSM Black DC863 0140106 PR RT
MSM Black DC880 0140626 PR RT
MSM Black DC885a 0140902 PR RT
MSM Black DC892a 0131101 PR RT
MSM Black DC897a 0140418 PR RT
MSM Black DC907 0140604 PR RT
MSM Black DC914 0140424 PR RT
MSM Black DC918a 0140908 PR RT
MSM Black DC920 0140519 PR RT
MSM Black DC921 0140821 PR RT
MSM Black DC930 0150116 PR RT IN
MSM Black DC944 0150417 IN
MSM Black DC958 0120515 PR RT
MSM Black DC959 0110526 PR RT
MSM Black DC977a 0110728 PR RT
MSM Black DC98a 0110906 PR RT
MSM Black DC996a 0130418 IN
MSM Black DC999 0140116 PR RT
MSM Hispa DC1065a 0121102 PR RT
MSM Hispa DC1291 0120831 PR RT
MSM Hispa DC1349 0120224 PR RT
MSM Hispa DC1377 0130813 PR RT
MSM Hispa DC1428a 0121112 PR RT
MSM Hispa DC1443 0130607 PR RT
MSM Hispa DC1463a 0110421 PR RT
MSM Hispa DC1569 0120112 PR RT
MSM Hispa DC1639 0140806 PR RT
MSM Hispa DC1676a 0110616 PR RT
MSM Hispa DC1683 0111220 PR RT
MSM Hispa DC1760 0130809 PR RT
MSM Hispa DC1811a 0111014 PR RT
MSM Hispa DC1827a 0120124 PR RT
MSM Hispa DC1832 0120307 PR RT
MSM Hispa DC1862 0120106 PR RT
MSM Hispa DC1871 0110421 PR RT
MSM Hispa DC1872 0120906 PR RT
MSM Hispa DC200 0150501 PR RT IN
MSM Hispa DC550 0140514 PR RT
MSM Hispa DC662a 0150422 PR RT
MSM Hispa DC702 0120716 PR RT
MSM Hispa DC705 0120907 PR RT
MSM Hispa DC713 0121025 PR RT
MSM Hispa DC715 0121116 PR RT
MSM Hispa DC757 0111020 PR RT
MSM Hispa DC770 0120319 PR RT
MSM Hispa DC784 0130507 PR RT
MSM Hispa DC828 0130805 PR RT
MSM Hispa DC878 0140408 PR RT
MSM Hispa DC928 0150128 PR RT
MSM Hispa DC938 0150505 PR RT
MSM Hispa DC940 0150512 PR RT
MSM Other DC1627 0110422 PR RT
MSM Other DC1673 0110427 PR RT
MSM Other DC1748 0110603 PR RT
MSM Other DC1754 0120508 PR RT
MSM Other DC610 0120402 PR RT
MSM Other DC752 0121207 PR RT
MSM Other DC765 0120320 PR RT
MSM Other DC871 0110822 PR RT
MSM Unkno DC1131 0111228 PR RT
MSM Unkno DC1293a 0110223 PR RT
MSM Unkno DC1572a 0110211 PR RT
MSM Unkno DC1610a 0131219 PR RT
MSM Unkno DC1630 0110127 PR RT
MSM Unkno DC1779 0110509 PR RT
MSM Unkno DC302a 0120411 PR RT
MSM Unkno DC500a 0110414 PR RT
MSM Unkno DC883 0140317 PR RT
MSM Unkno DC957 0150505 PR RT IN
MSM White DC1035a 0110829 PR RT
MSM White DC1070 0120124 PR RT
MSM White DC109 0110120 PR RT
MSM White DC1169 0120227 PR RT
MSM White DC1188a 0120911 PR RT
MSM White DC120 0130820 PR RT
MSM White DC1254a 0120430 PR RT
MSM White DC1284 0110128 PR RT
MSM White DC1352 0140113 PR RT
MSM White DC1364 0130912 PR RT
MSM White DC138 0120711 PR RT
MSM White DC1380 0130529 PR RT
MSM White DC1420 0120109 PR RT
MSM White DC147 0130423 PR RT
MSM White DC1483 0150210 PR RT
MSM White DC1497 0110208 PR RT
MSM White DC1498 0150113 PR RT
MSM White DC1499 0130709 PR RT
MSM White DC1517a 0110413 PR RT
MSM White DC1546 0150126 PR RT

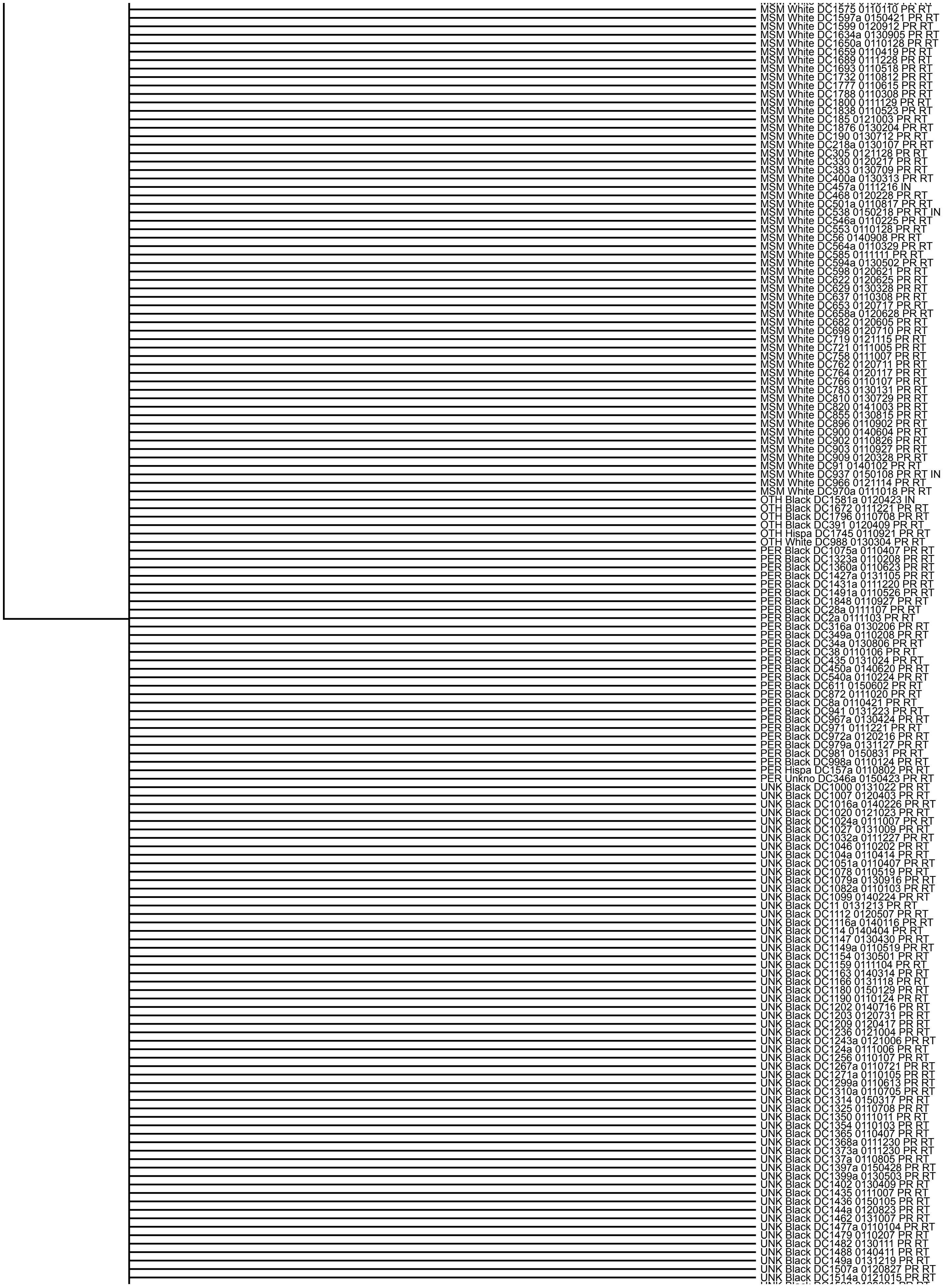
MSM White DC1575 0110110 PR RT
MSM White DC1597a 0150421 PR RT
MSM White DC1599 0120912 PR RT
MSM White DC1634a 0130905 PR RT
MSM White DC1650a 0110128 PR RT
MSM White DC1659 0110419 PR RT
MSM White DC1689 0111228 PR RT
MSM White DC1693 0110518 PR RT
MSM White DC1732 0110812 PR RT
MSM White DC1777 0110615 PR RT
MSM White DC1788 0110308 PR RT
MSM White DC1800 0111129 PR RT
MSM White DC1838 0110523 PR RT
MSM White DC185 0121003 PR RT
MSM White DC1876 0130204 PR RT
MSM White DC190 0130712 PR RT
MSM White DC218a 0130107 PR RT
MSM White DC305 0121128 PR RT
MSM White DC330 0120217 PR RT
MSM White DC383 0130709 PR RT
MSM White DC400a 0130313 PR RT
MSM White DC457a 0111216 IN
MSM White DC468 0120228 PR RT
MSM White DC501a 0110817 PR RT
MSM White DC538 0150218 PR RT IN
MSM White DC546a 0110225 PR RT
MSM White DC553 0110128 PR RT
MSM White DC56 0140908 PR RT
MSM White DC564a 0110329 PR RT
MSM White DC585 011111 PR RT
MSM White DC594a 0130502 PR RT
MSM White DC598 0120621 PR RT
MSM White DC622 0120625 PR RT
MSM White DC629 0130328 PR RT
MSM White DC637 0110308 PR RT
MSM White DC653 0120717 PR RT
MSM White DC658a 0120628 PR RT
MSM White DC682 0120605 PR RT
MSM White DC698 0120710 PR RT
MSM White DC719 0121115 PR RT
MSM White DC721 0111005 PR RT
MSM White DC758 0111007 PR RT
MSM White DC762 0120711 PR RT
MSM White DC764 0120117 PR RT
MSM White DC766 0110107 PR RT
MSM White DC783 0130131 PR RT
MSM White DC810 0130729 PR RT
MSM White DC820 0141003 PR RT
MSM White DC855 0130815 PR RT
MSM White DC896 0110902 PR RT
MSM White DC900 0140604 PR RT
MSM White DC902 0110826 PR RT
MSM White DC903 0110927 PR RT
MSM White DC909 0120328 PR RT
MSM White DC91 0140102 PR RT
MSM White DC937 0150108 PR RT IN
MSM White DC966 0121114 PR RT
MSM White DC970a 0111018 PR RT
OTH Black DC1581a 0120423 IN
OTH Black DC1672 0111221 PR RT
OTH Black DC1796 0110708 PR RT
OTH Black DC391 0120409 PR RT
OTH Hispa DC1745 0110921 PR RT
OTH White DC988 0130304 PR RT
PER Black DC1075a 0110407 PR RT
PER Black DC1323a 0110208 PR RT
PER Black DC1360a 0110623 PR RT
PER Black DC1427a 0131105 PR RT
PER Black DC1431a 0111220 PR RT
PER Black DC1491a 0110526 PR RT
PER Black DC1848 0110927 PR RT
PER Black DC28a 0111107 PR RT
PER Black DC2a 0111103 PR RT
PER Black DC316a 0130206 PR RT
PER Black DC349a 0110208 PR RT
PER Black DC34a 0130806 PR RT
PER Black DC38 0110106 PR RT
PER Black DC435 0131024 PR RT
PER Black DC450a 0140620 PR RT
PER Black DC540a 0110224 PR RT
PER Black DC611 0150602 PR RT
PER Black DC872 0111020 PR RT
PER Black DC8a 0110421 PR RT
PER Black DC941 0131223 PR RT
PER Black DC967a 0130424 PR RT
PER Black DC971 0111221 PR RT
PER Black DC972a 0120216 PR RT
PER Black DC979a 0131127 PR RT
PER Black DC981 0150831 PR RT
PER Black DC998a 0110124 PR RT
PER Hispa DC157a 0110802 PR RT
PER Unkno DC346a 0150423 PR RT
UNK Black DC1000 0131022 PR RT
UNK Black DC1007 0120403 PR RT
UNK Black DC1016a 0140226 PR RT
UNK Black DC1020 0121023 PR RT
UNK Black DC1024a 0111007 PR RT
UNK Black DC1027 0131009 PR RT
UNK Black DC1032a 0111227 PR RT
UNK Black DC1046 0110202 PR RT
UNK Black DC104a 0110414 PR RT
UNK Black DC1051a 0110407 PR RT
UNK Black DC1078 0110519 PR RT
UNK Black DC1079a 0130916 PR RT
UNK Black DC1082a 0110103 PR RT
UNK Black DC1099 0140224 PR RT
UNK Black DC11 0131213 PR RT
UNK Black DC1112 0120507 PR RT
UNK Black DC1116a 0140116 PR RT
UNK Black DC114 0140404 PR RT
UNK Black DC1147 0130430 PR RT
UNK Black DC1149a 0110519 PR RT
UNK Black DC1154 0130501 PR RT
UNK Black DC1159 0111104 PR RT
UNK Black DC1163 0140314 PR RT
UNK Black DC1166 0131118 PR RT
UNK Black DC1180 0150129 PR RT
UNK Black DC1190 0110124 PR RT
UNK Black DC1202 0140716 PR RT
UNK Black DC1203 0120731 PR RT
UNK Black DC1209 0120417 PR RT
UNK Black DC1236 0121004 PR RT
UNK Black DC1243a 0121006 PR RT
UNK Black DC124a 0111006 PR RT
UNK Black DC1256 0110107 PR RT
UNK Black DC1267a 0110721 PR RT
UNK Black DC1271a 0110105 PR RT
UNK Black DC1299a 0110613 PR RT
UNK Black DC1310a 0110705 PR RT
UNK Black DC1314 0150317 PR RT
UNK Black DC1325 0110708 PR RT
UNK Black DC1350 0111011 PR RT
UNK Black DC1354 0110103 PR RT
UNK Black DC1365 0110407 PR RT
UNK Black DC1368a 0111230 PR RT
UNK Black DC1373a 0111230 PR RT
UNK Black DC137a 0110805 PR RT
UNK Black DC1397a 0150428 PR RT
UNK Black DC1399a 0130503 PR RT
UNK Black DC1402 0130409 PR RT
UNK Black DC1435 0111007 PR RT
UNK Black DC1436 0150105 PR RT
UNK Black DC144a 0120823 PR RT
UNK Black DC1462 0131007 PR RT
UNK Black DC1477a 0110104 PR RT
UNK Black DC1479 0110207 PR RT
UNK Black DC1482 0130111 PR RT
UNK Black DC1488 0140411 PR RT
UNK Black DC149a 0131219 PR RT
UNK Black DC1507a 0120827 PR RT
UNK Black DC1514a 0121015 PR RT

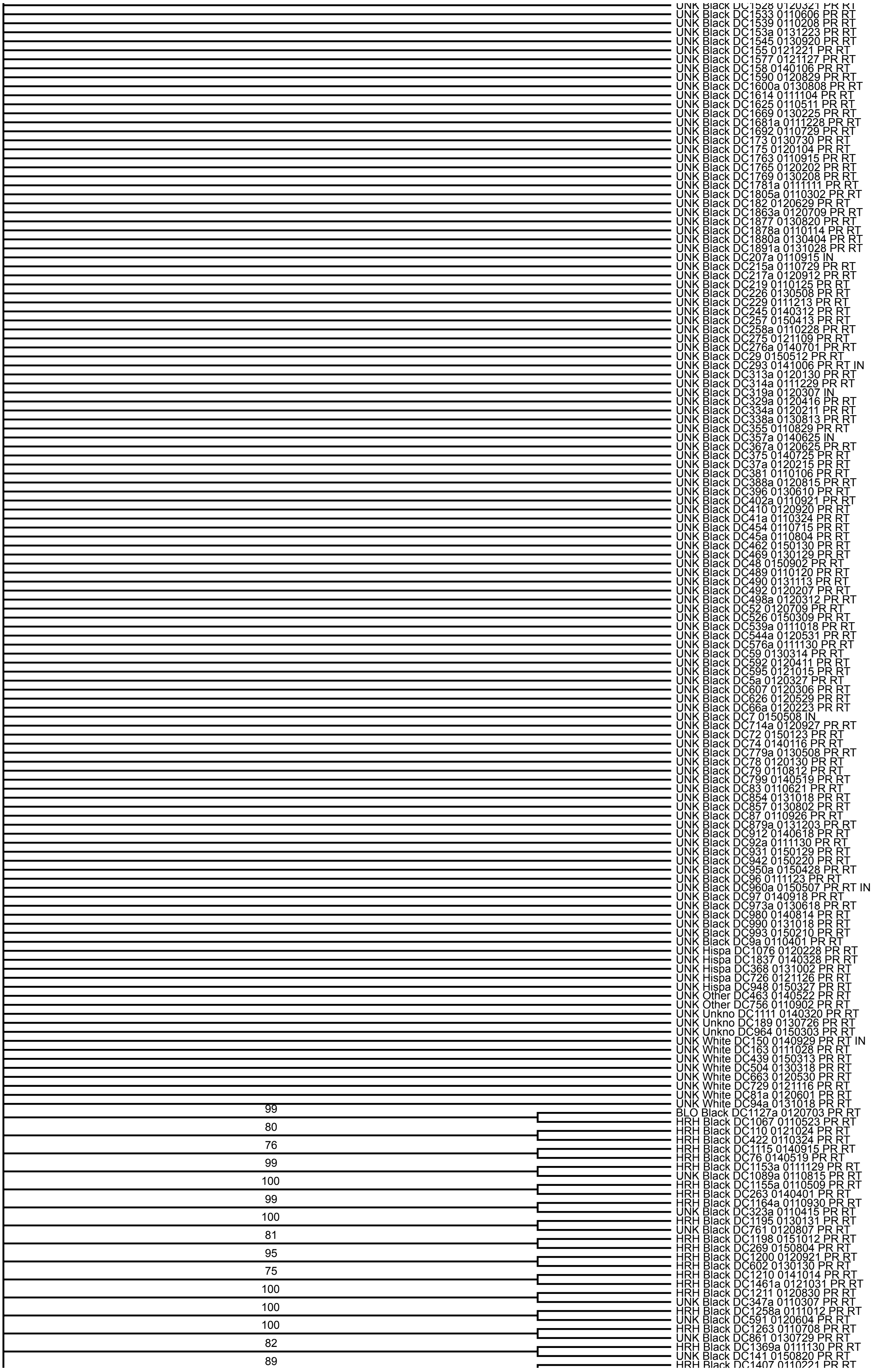

UNK Black DC1528 0120321 PR RT
UNK Black DC1533 0110606 PR RT
UNK Black DC1539 0110208 PR RT
UNK Black DC153a 0131223 PR RT
UNK Black DC1545 0130920 PR RT
UNK Black DC155 0121221 PR RT
UNK Black DC1577 0121127 PR RT
UNK Black DC158 0140106 PR RT
UNK Black DC1590 0120829 PR RT
UNK Black DC1600a 0130808 PR RT
UNK Black DC1614 0111104 PR RT
UNK Black DC1625 0110511 PR RT
UNK Black DC1669 0130225 PR RT
UNK Black DC1681a 0111228 PR RT
UNK Black DC1692 0110729 PR RT
UNK Black DC173 0130730 PR RT
UNK Black DC175 0120104 PR RT
UNK Black DC1763 0110915 PR RT
UNK Black DC1765 0120202 PR RT
UNK Black DC1769 0130208 PR RT
UNK Black DC1781a 0111111 PR RT
UNK Black DC1805a 0110302 PR RT
UNK Black DC182 0120629 PR RT
UNK Black DC1863a 0120709 PR RT
UNK Black DC1877 0130820 PR RT
UNK Black DC1878a 0110114 PR RT
UNK Black DC1880a 0130404 PR RT
UNK Black DC1891a 0131028 PR RT
UNK Black DC207a 0110915 IN
UNK Black DC215a 0110729 PR RT
UNK Black DC217a 0120912 PR RT
UNK Black DC219 0110125 PR RT
UNK Black DC226 0130508 PR RT
UNK Black DC229 0111213 PR RT
UNK Black DC245 0140312 PR RT
UNK Black DC257 0150413 PR RT
UNK Black DC258a 0110228 PR RT
UNK Black DC275 0121109 PR RT
UNK Black DC276a 0140701 PR RT
UNK Black DC29 0150512 PR RT
UNK Black DC293 0141006 PR RT IN
UNK Black DC313a 0120130 PR RT
UNK Black DC314a 0111229 PR RT
UNK Black DC319a 0120307 IN
UNK Black DC329a 0120416 PR RT
UNK Black DC334a 0120211 PR RT
UNK Black DC338a 0130813 PR RT
UNK Black DC355 0110829 PR RT
UNK Black DC357a 0140625 IN
UNK Black DC367a 0120625 PR RT
UNK Black DC375 0140725 PR RT
UNK Black DC37a 0120215 PR RT
UNK Black DC381 0110106 PR RT
UNK Black DC388a 0120815 PR RT
UNK Black DC396 0130610 PR RT
UNK Black DC402a 0110921 PR RT
UNK Black DC410 0120920 PR RT
UNK Black DC41a 0110324 PR RT
UNK Black DC454 0110715 PR RT
UNK Black DC45a 0110804 PR RT
UNK Black DC462 0150130 PR RT
UNK Black DC469 0130129 PR RT
UNK Black DC48 0150902 PR RT
UNK Black DC489 0110120 PR RT
UNK Black DC490 0131113 PR RT
UNK Black DC492 0120207 PR RT
UNK Black DC498a 0120312 PR RT
UNK Black DC52 0120709 PR RT
UNK Black DC526 0150309 PR RT
UNK Black DC539a 0111018 PR RT
UNK Black DC544a 0120531 PR RT
UNK Black DC576a 0111130 PR RT
UNK Black DC59 0130314 PR RT
UNK Black DC592 0120411 PR RT
UNK Black DC595 0121015 PR RT
UNK Black DC5a 0120327 PR RT
UNK Black DC607 0120306 PR RT
UNK Black DC626 0120529 PR RT
UNK Black DC66a 0120223 PR RT
UNK Black DC7 0150508 IN
UNK Black DC714a 0120927 PR RT
UNK Black DC72 0150123 PR RT
UNK Black DC74 0140116 PR RT
UNK Black DC779a 0130508 PR RT
UNK Black DC78 0120130 PR RT
UNK Black DC79 0110812 PR RT
UNK Black DC799 0140519 PR RT
UNK Black DC83 0110621 PR RT
UNK Black DC854 0131018 PR RT
UNK Black DC857 0130802 PR RT
UNK Black DC87 0110926 PR RT
UNK Black DC879a 0131203 PR RT
UNK Black DC912 0140618 PR RT
UNK Black DC92a 0111130 PR RT
UNK Black DC931 0150129 PR RT
UNK Black DC942 0150220 PR RT
UNK Black DC950a 0150428 PR RT
UNK Black DC96 0111123 PR RT
UNK Black DC960a 0150507 PR RT IN
UNK Black DC97 0140918 PR RT
UNK Black DC973a 0130618 PR RT
UNK Black DC980 0140814 PR RT
UNK Black DC990 0131018 PR RT
UNK Black DC993 0150210 PR RT
UNK Black DC9a 0110401 PR RT
UNK Hispa DC1076 0120228 PR RT
UNK Hispa DC1837 0140328 PR RT
UNK Hispa DC368 0131002 PR RT
UNK Hispa DC726 0121126 PR RT
UNK Hispa DC948 0150327 PR RT
UNK Other DC463 0140522 PR RT
UNK Other DC756 0110902 PR RT
UNK Unkno DC1111 0140320 PR RT
UNK Unkno DC189 0130726 PR RT
UNK Unkno DC964 0150303 PR RT
UNK White DC150 0140929 PR RT IN
UNK White DC163 0111028 PR RT
UNK White DC439 0150313 PR RT
UNK White DC504 0130318 PR RT
UNK White DC663 0120530 PR RT
UNK White DC729 0121116 PR RT
UNK White DC81a 0120601 PR RT
UNK White DC94a 0131018 PR RT
99
BLO Black DC1127a 0120703 PR RT
HRH Black DC1067 0110523 PR RT
80
HRH Black DC110 0121024 PR RT
HRH Black DC422 0110324 PR RT
76
HRH Black DC1115 0140915 PR RT
HRH Black DC76 0140519 PR RT
99
HRH Black DC1153a 0111129 PR RT
UNK Black DC1089a 0110815 PR RT
100
HRH Black DC1155a 0110509 PR RT
HRH Black DC263 0140401 PR RT
99
HRH Black DC1164a 0110930 PR RT
UNK Black DC323a 0110415 PR RT
100
HRH Black DC1195 0130131 PR RT
UNK Black DC761 0120807 PR RT
81
HRH Black DC1198 0151012 PR RT
HRH Black DC269 0150804 PR RT
95
HRH Black DC1200 0120921 PR RT
HRH Black DC602 0130130 PR RT
75
HRH Black DC1210 0141014 PR RT
HRH Black DC1461a 0121031 PR RT
100
HRH Black DC1211 0120830 PR RT
UNK Black DC347a 0110307 PR RT
100
HRH Black DC1258a 0111012 PR RT
UNK Black DC591 0120604 PR RT
100
HRH Black DC1263 0110708 PR RT
UNK Black DC861 0130729 PR RT
82
HRH Black DC1369a 0111130 PR RT
UNK Black DC141 0150820 PR RT
89
HRH Black DC1407 0110221 PR RT

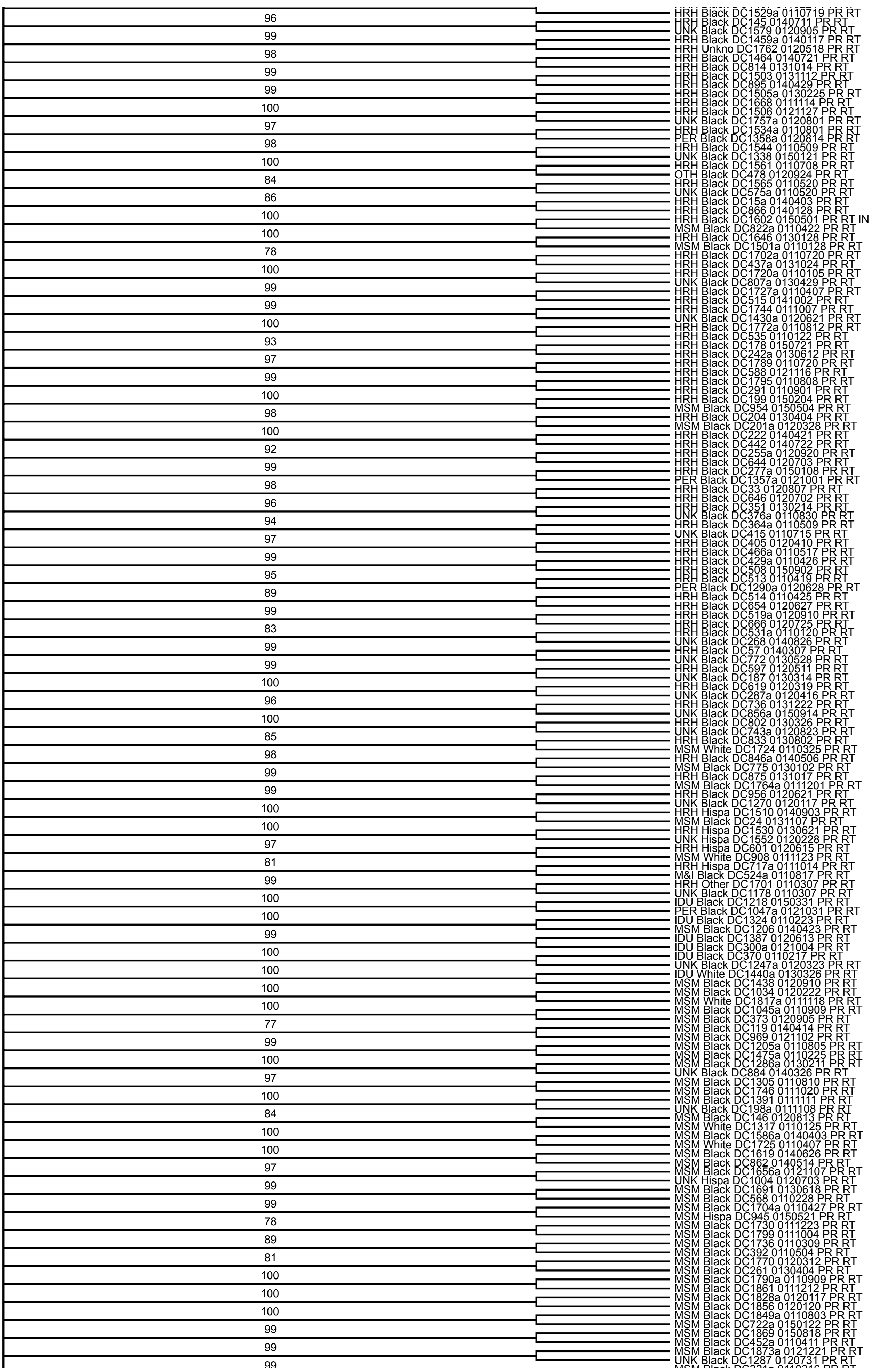

HRH Black DC1529a 0110719 PR RT
96
HRH Black DC145 0140711 PR RT
UNK Black DC1579 0120905 PR RT
99
HRH Black DC1459a 0140117 PR RT
HRH Unkno DC1762 0120518 PR RT
98
HRH Black DC1464 0140721 PR RT
HRH Black DC814 0131014 PR RT
99
HRH Black DC1503 0131112 PR RT
HRH Black DC895 0140429 PR RT
99
HRH Black DC1505a 0130225 PR RT
HRH Black DC1668 0111114 PR RT
100
HRH Black DC1506 0121127 PR RT
UNK Black DC1757a 0120801 PR RT
97
HRH Black DC1534a 0110801 PR RT
PER Black DC1358a 0120814 PR RT
98
HRH Black DC1544 0110509 PR RT
UNK Black DC1338 0150121 PR RT
100
HRH Black DC1561 0110708 PR RT
OTH Black DC478 0120924 PR RT
84
HRH Black DC1565 0110520 PR RT
UNK Black DC575a 0110520 PR RT
86
HRH Black DC15a 0140403 PR RT
HRH Black DC866 0140128 PR RT
100
HRH Black DC1602 0150501 PR RT IN
MSM Black DC822a 0110422 PR RT
100
HRH Black DC1646 0130128 PR RT
MSM Black DC1501a 0110128 PR RT
78
HRH Black DC1702a 0110720 PR RT
HRH Black DC437a 0131024 PR RT
100
HRH Black DC1720a 0110105 PR RT
UNK Black DC807a 0130429 PR RT
99
HRH Black DC1727a 0110407 PR RT
HRH Black DC515 0141002 PR RT
99
HRH Black DC1744 0111007 PR RT
UNK Black DC1430a 0120621 PR RT
100
HRH Black DC1772a 0110812 PR RT
HRH Black DC535 0110122 PR RT
93
HRH Black DC178 0150721 PR RT
HRH Black DC242a 0130612 PR RT
97
HRH Black DC1789 0110720 PR RT
HRH Black DC588 0121116 PR RT
99
HRH Black DC1795 0110808 PR RT
HRH Black DC291 0110901 PR RT
100
HRH Black DC199 0150204 PR RT
MSM Black DC954 0150504 PR RT
98
HRH Black DC204 0130404 PR RT
MSM Black DC201a 0120328 PR RT
100
HRH Black DC222 0140421 PR RT
HRH Black DC442 0140722 PR RT
92
HRH Black DC255a 0120920 PR RT
HRH Black DC644 0120703 PR RT
99
HRH Black DC277a 0150108 PR RT
PER Black DC1357a 0121001 PR RT
98
HRH Black DC33 0120807 PR RT
HRH Black DC646 0120702 PR RT
96
HRH Black DC351 0130214 PR RT
UNK Black DC376a 0110830 PR RT
94
HRH Black DC364a 0110509 PR RT
UNK Black DC415 0110715 PR RT
97
HRH Black DC405 0120410 PR RT
HRH Black DC466a 0110517 PR RT
99
HRH Black DC429a 0110426 PR RT
HRH Black DC508 0150902 PR RT
95
HRH Black DC513 0110419 PR RT
PER Black DC1290a 0120628 PR RT
89
HRH Black DC514 0110425 PR RT
HRH Black DC654 0120627 PR RT
99
HRH Black DC519a 0120910 PR RT
HRH Black DC666 0120725 PR RT
83
HRH Black DC531a 0110120 PR RT
UNK Black DC268 0140826 PR RT
99
HRH Black DC57 0140307 PR RT
UNK Black DC772 0130528 PR RT
99
HRH Black DC597 0120511 PR RT
UNK Black DC187 0130314 PR RT
100
HRH Black DC619 0120319 PR RT
UNK Black DC287a 0120416 PR RT
96
HRH Black DC736 0131222 PR RT
UNK Black DC856a 0150914 PR RT
100
HRH Black DC802 0130326 PR RT
UNK Black DC743a 0120823 PR RT
85
HRH Black DC833 0130802 PR RT
MSM White DC1724 0110325 PR RT
98
HRH Black DC846a 0140506 PR RT
MSM Black DC775 0130102 PR RT
99
HRH Black DC875 0131017 PR RT
MSM Black DC1764a 0111201 PR RT
99
HRH Black DC956 0120621 PR RT
UNK Black DC1270 0120117 PR RT
100
HRH Hispa DC1510 0140903 PR RT
MSM Black DC24 0131107 PR RT
100
HRH Hispa DC1530 0130621 PR RT
UNK Hispa DC1552 0120228 PR RT
97
HRH Hispa DC601 0120615 PR RT
MSM White DC908 0111123 PR RT
81
HRH Hispa DC717a 0111014 PR RT
M&I Black DC524a 0110817 PR RT
99
HRH Other DC1701 0110307 PR RT
UNK Black DC1178 0110307 PR RT
100
IDU Black DC1218 0150331 PR RT
PER Black DC1047a 0121031 PR RT
100
IDU Black DC1324 0110223 PR RT
MSM Black DC1206 0140423 PR RT
99
IDU Black DC1387 0120613 PR RT
IDU Black DC300a 0121004 PR RT
100
IDU Black DC370 0110217 PR RT
UNK Black DC1247a 0120323 PR RT
100
IDU White DC1440a 0130326 PR RT
MSM Black DC1438 0120910 PR RT
100
MSM Black DC1034 0120222 PR RT
MSM White DC1817a 0111118 PR RT
100
MSM Black DC1045a 0110909 PR RT
MSM Black DC373 0120905 PR RT
77
MSM Black DC119 0140414 PR RT
MSM Black DC969 0121102 PR RT
99
MSM Black DC1205a 0110805 PR RT
MSM Black DC1475a 0110225 PR RT
100
MSM Black DC1286a 0130211 PR RT
UNK Black DC884 0140326 PR RT
97
MSM Black DC1305 0110810 PR RT
MSM Black DC1746 0111020 PR RT
100
MSM Black DC1391 0111111 PR RT
UNK Black DC198a 0111108 PR RT
84
MSM Black DC146 0120813 PR RT
MSM White DC1317 0110125 PR RT
100
MSM Black DC1586a 0140403 PR RT
MSM White DC1725 0110407 PR RT
100
MSM Black DC1619 0140626 PR RT
MSM Black DC862 0140514 PR RT
97
MSM Black DC1656a 0121107 PR RT
UNK Hispa DC1004 0120703 PR RT
99
MSM Black DC1691 0130618 PR RT
MSM Black DC568 0110228 PR RT
99
MSM Black DC1704a 0110427 PR RT
MSM Hispa DC945 0150521 PR RT
78
MSM Black DC1730 0111223 PR RT
MSM Black DC1799 0111004 PR RT
89
MSM Black DC1736 0110309 PR RT
MSM Black DC392 0110504 PR RT
81
MSM Black DC1770 0120312 PR RT
MSM Black DC261 0130404 PR RT
100
MSM Black DC1790a 0110909 PR RT
MSM Black DC1861 0111212 PR RT
100
MSM Black DC1828a 0120117 PR RT
MSM Black DC1856 0120120 PR RT
100
MSM Black DC1849a 0110803 PR RT
MSM Black DC722a 0150122 PR RT
99
MSM Black DC1869 0150818 PR RT
MSM Black DC452a 0110411 PR RT
99
MSM Black DC1873a 0121221 PR RT
UNK Black DC1287 0120731 PR RT
99

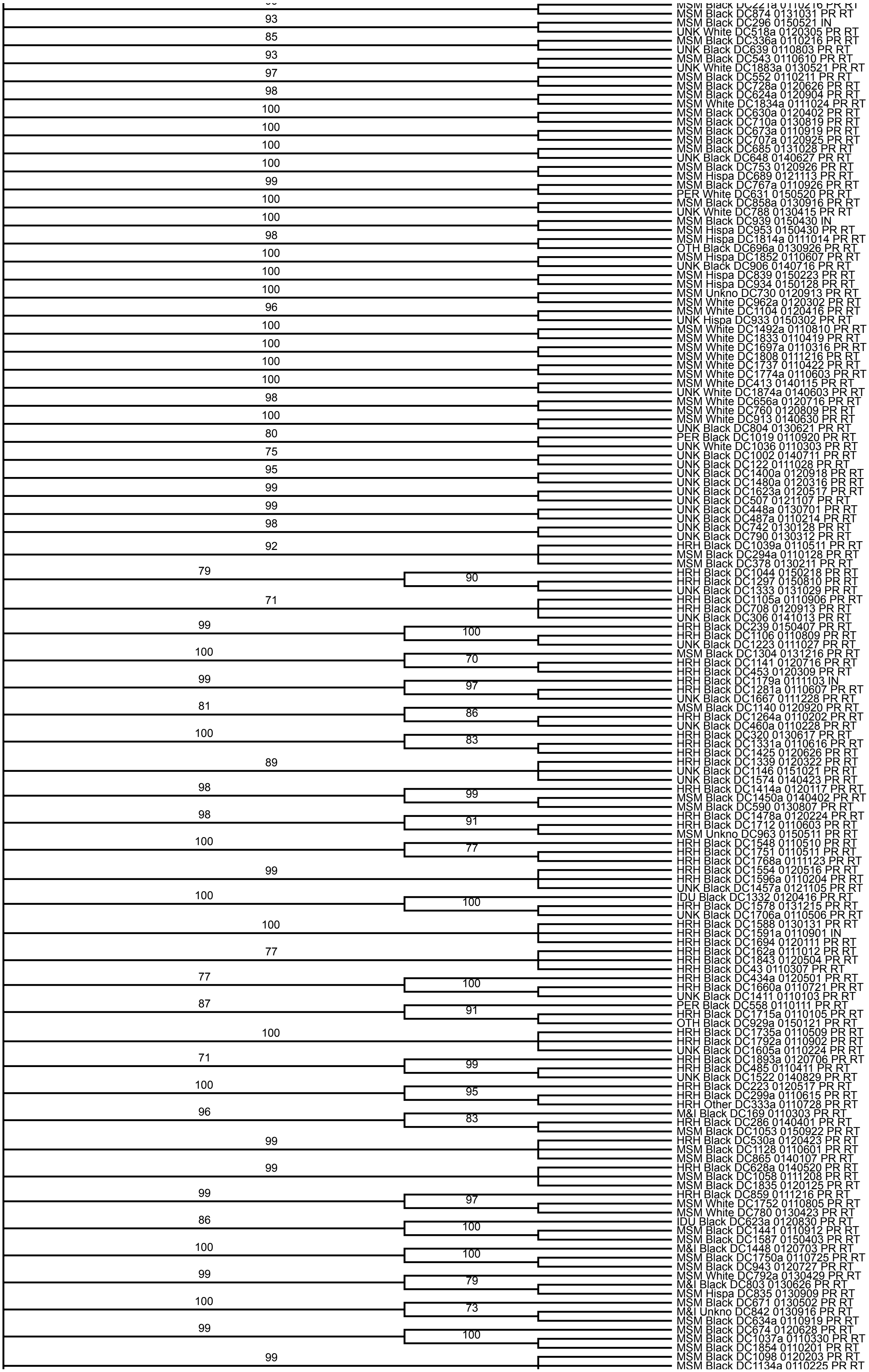

MSM Black DC874 0131031 PR RT
MSM Black DC296 0150521 IN
UNK White DC518a 0120305 PR RT
MSM Black DC336a 0110216 PR RT
UNK Black DC639 0110803 PR RT
MSM Black DC543 0110610 PR RT
UNK White DC1883a 0130521 PR RT
MSM Black DC552 0110211 PR RT
MSM Black DC728a 0120626 PR RT
MSM Black DC624a 0120904 PR RT
MSM White DC1834a 0111024 PR RT
MSM Black DC630a 0120402 PR RT
MSM Black DC710a 0130819 PR RT
MSM Black DC673a 0110919 PR RT
MSM Black DC707a 0120925 PR RT
MSM Black DC685 0131028 PR RT
UNK Black DC648 0140627 PR RT
MSM Black DC753 0120926 PR RT
MSM Hispa DC689 0121113 PR RT
MSM Black DC767a 0110926 PR RT
PER White DC631 0150520 PR RT
MSM Black DC858a 0130916 PR RT
UNK White DC788 0130415 PR RT
MSM Black DC939 0150430 IN
MSM Hispa DC953 0150430 PR RT
MSM Hispa DC1814a 0111014 PR RT
OTH Black DC696a 0130926 PR RT
MSM Hispa DC1852 0110607 PR RT
UNK Black DC906 0140716 PR RT
MSM Hispa DC839 0150223 PR RT
MSM Hispa DC934 0150128 PR RT
MSM Unkno DC730 0120913 PR RT
MSM White DC962a 0120302 PR RT
MSM White DC1104 0120416 PR RT
UNK Hispa DC933 0150302 PR RT
MSM White DC1492a 0110810 PR RT
MSM White DC1833 0110419 PR RT
MSM White DC1697a 0110316 PR RT
MSM White DC1808 0111216 PR RT
MSM White DC1737 0110422 PR RT
MSM White DC1774a 0110603 PR RT
MSM White DC413 0140115 PR RT
UNK White DC1874a 0140603 PR RT
MSM White DC656a 0120716 PR RT
MSM White DC760 0120809 PR RT
MSM White DC913 0140630 PR RT
UNK Black DC804 0130621 PR RT
PER Black DC1019 0110920 PR RT
UNK White DC1036 0110303 PR RT
UNK Black DC1002 0140711 PR RT
UNK Black DC122 0111028 PR RT
UNK Black DC1400a 0120918 PR RT
UNK Black DC1480a 0120316 PR RT
UNK Black DC1623a 0120517 PR RT
UNK Black DC507 0121107 PR RT
UNK Black DC448a 0130701 PR RT
UNK Black DC487a 0110214 PR RT
UNK Black DC742 0130128 PR RT
UNK Black DC790 0130312 PR RT
HRH Black DC1039a 0110511 PR RT
MSM Black DC294a 0110128 PR RT
MSM Black DC378 0130211 PR RT
HRH Black DC1044 0150218 PR RT
HRH Black DC1297 0150810 PR RT
UNK Black DC1333 0131029 PR RT
HRH Black DC1105a 0110906 PR RT
HRH Black DC708 0120913 PR RT
UNK Black DC306 0141013 PR RT
HRH Black DC239 0150407 PR RT
HRH Black DC1106 0110809 PR RT
UNK Black DC1223 0111027 PR RT
MSM Black DC1304 0131216 PR RT
HRH Black DC1141 0120716 PR RT
HRH Black DC453 0120309 PR RT
HRH Black DC1179a 0111103 IN
HRH Black DC1281a 0110607 PR RT
UNK Black DC1667 0111228 PR RT
MSM Black DC1140 0120920 PR RT
HRH Black DC1264a 0110202 PR RT
UNK Black DC460a 0110228 PR RT
HRH Black DC320 0130617 PR RT
HRH Black DC1331a 0110616 PR RT
HRH Black DC1425 0120626 PR RT
HRH Black DC1339 0120322 PR RT
UNK Black DC1146 0151021 PR RT
UNK Black DC1574 0140423 PR RT
HRH Black DC1414a 0120117 PR RT
MSM Black DC1450a 0140402 PR RT
MSM Black DC590 0130807 PR RT
HRH Black DC1478a 0120224 PR RT
HRH Black DC1712 0110603 PR RT
MSM Unkno DC963 0150511 PR RT
HRH Black DC1548 0110510 PR RT
HRH Black DC1751 0110511 PR RT
HRH Black DC1768a 0111123 PR RT
HRH Black DC1554 0120516 PR RT
HRH Black DC1596a 0110204 PR RT
UNK Black DC1457a 0121105 PR RT
IDU Black DC1332 0120416 PR RT
HRH Black DC1578 0131215 PR RT
UNK Black DC1706a 0110506 PR RT
HRH Black DC1588 0130131 PR RT
HRH Black DC1591a 0110901 IN
HRH Black DC1694 0120111 PR RT
HRH Black DC162a 0111012 PR RT
HRH Black DC1843 0120504 PR RT
HRH Black DC43 0110307 PR RT
HRH Black DC434a 0120501 PR RT
HRH Black DC1660a 0110721 PR RT
UNK Black DC1411 0110103 PR RT
PER Black DC558 0110111 PR RT
HRH Black DC1715a 0110105 PR RT
OTH Black DC929a 0150121 PR RT
HRH Black DC1735a 0110509 PR RT
HRH Black DC1792a 0110902 PR RT
UNK Black DC1605a 0110224 PR RT
HRH Black DC1893a 0120706 PR RT
HRH Black DC485 0110411 PR RT
UNK Black DC1522 0140829 PR RT
HRH Black DC223 0120517 PR RT
HRH Black DC299a 0110615 PR RT
HRH Other DC333a 0110728 PR RT
M&I Black DC169 0110303 PR RT
HRH Black DC286 0140401 PR RT
MSM Black DC1053 0150922 PR RT
HRH Black DC530a 0120423 PR RT
MSM Black DC1128 0110601 PR RT
MSM Black DC865 0140107 PR RT
HRH Black DC628a 0140520 PR RT
MSM Black DC1058 0111208 PR RT
MSM Black DC1835 0120125 PR RT
HRH Black DC859 0111216 PR RT
MSM White DC1752 0110805 PR RT
MSM White DC780 0130423 PR RT
IDU Black DC623a 0120830 PR RT
MSM Black DC1441 0110912 PR RT
MSM Black DC1587 0150403 PR RT
M&I Black DC1448 0120703 PR RT
MSM Black DC1750a 0110725 PR RT
MSM Black DC943 0120727 PR RT
MSM White DC792a 0130429 PR RT
M&I Black DC803 0130626 PR RT
MSM Hispa DC835 0130909 PR RT
MSM Black DC671 0130502 PR RT
M&I Unkno DC842 0130916 PR RT
MSM Black DC634a 0110919 PR RT
MSM Black DC674 0120628 PR RT
MSM Black DC1037a 0110330 PR RT
MSM Black DC1854 0110201 PR RT
MSM Black DC1098 0120203 PR RT
MSM Black DC1134a 0110225 PR RT

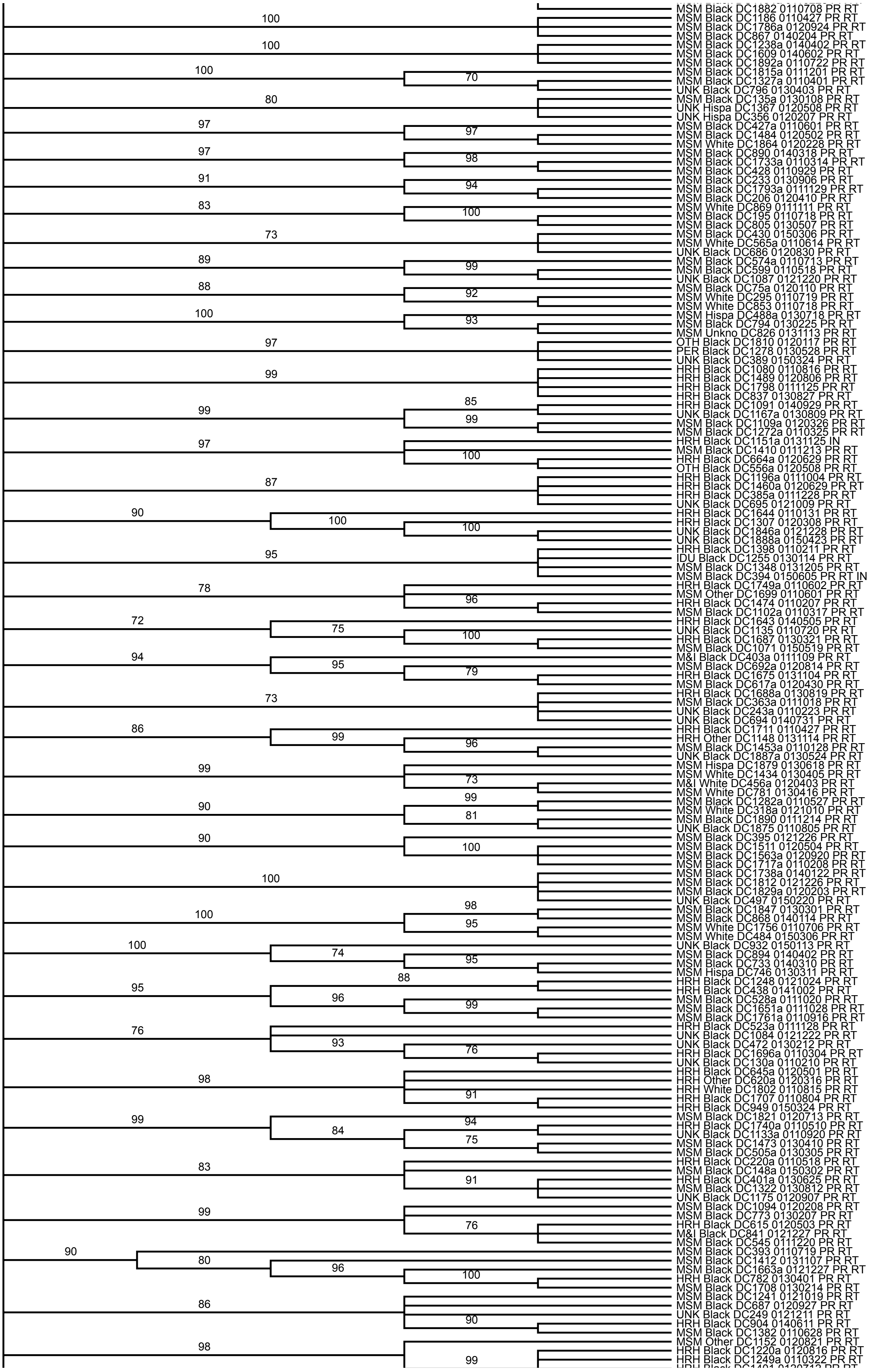
MSM Black DC1882 0110708 PR RT
MSM Black DC1186 0110427 PR RT
MSM Black DC1786a 0120924 PR RT
MSM Black DC867 0140204 PR RT
MSM Black DC1238a 0140402 PR RT
MSM Black DC1609 0140602 PR RT
MSM Black DC1892a 0110722 PR RT
MSM Black DC1815a 0111201 PR RT
MSM Black DC1327a 0110401 PR RT
UNK Black DC796 0130403 PR RT
MSM Black DC135a 0130108 PR RT
UNK Hispa DC1367 0120508 PR RT
UNK Hispa DC356 0120207 PR RT
MSM Black DC427a 0110601 PR RT
MSM Black DC1484 0120502 PR RT
MSM White DC1864 0120228 PR RT
MSM Black DC890 0140318 PR RT
MSM Black DC1733a 0110314 PR RT
MSM Black DC428 0110929 PR RT
MSM Black DC233 0130906 PR RT
MSM Black DC1793a 0111129 PR RT
MSM Black DC206 0120410 PR RT
MSM White DC869 0111111 PR RT
MSM Black DC195 0110718 PR RT
MSM Black DC805 0130507 PR RT
MSM Black DC430 0150306 PR RT
MSM White DC565a 0110614 PR RT
UNK Black DC686 0120830 PR RT
MSM Black DC574a 0110713 PR RT
MSM Black DC599 0110518 PR RT
UNK Black DC1087 0121220 PR RT
MSM Black DC75a 0120110 PR RT
MSM White DC295 0110719 PR RT
MSM White DC853 0110718 PR RT
MSM Hispa DC488a 0130718 PR RT
MSM Black DC794 0130225 PR RT
MSM Unkno DC826 0131113 PR RT
OTH Black DC1810 0120117 PR RT
PER Black DC1278 0130528 PR RT
UNK Black DC389 0150324 PR RT
HRH Black DC1080 0110816 PR RT
HRH Black DC1489 0120806 PR RT
HRH Black DC1798 0111125 PR RT
HRH Black DC837 0130827 PR RT
HRH Black DC1091 0140929 PR RT
UNK Black DC1167a 0130809 PR RT
MSM Black DC1109a 0120326 PR RT
MSM Black DC1272a 0110325 PR RT
HRH Black DC1151a 0131125 IN
MSM Black DC1410 0111213 PR RT
HRH Black DC664a 0120629 PR RT
OTH Black DC556a 0120508 PR RT
HRH Black DC1196a 0111004 PR RT
HRH Black DC1460a 0120629 PR RT
HRH Black DC385a 0111228 PR RT
UNK Black DC695 0121009 PR RT
HRH Black DC1644 0110131 PR RT
HRH Black DC1307 0120308 PR RT
UNK Black DC1846a 0121228 PR RT
UNK Black DC1888a 0150423 PR RT
HRH Black DC1398 0110211 PR RT
IDU Black DC1255 0130114 PR RT
MSM Black DC1348 0131205 PR RT
MSM Black DC394 0150605 PR RT IN
HRH Black DC1749a 0110602 PR RT
MSM Other DC1699 0110601 PR RT
HRH Black DC1474 0110207 PR RT
MSM Black DC1102a 0110317 PR RT
HRH Black DC1643 0140505 PR RT
UNK Black DC1135 0110720 PR RT
HRH Black DC1687 0130321 PR RT
MSM Black DC1071 0150519 PR RT
M&I Black DC403a 0111109 PR RT
MSM Black DC692a 0120814 PR RT
HRH Black DC1675 0131104 PR RT
MSM Black DC617a 0120430 PR RT
HRH Black DC1688a 0130819 PR RT
MSM Black DC363a 0111018 PR RT
UNK Black DC243a 0110223 PR RT
UNK Black DC694 0140731 PR RT
HRH Black DC1711 0110427 PR RT
HRH Other DC1148 0131114 PR RT
MSM Black DC1453a 0110128 PR RT
UNK Black DC1887a 0130524 PR RT
MSM Hispa DC1879 0130618 PR RT
MSM White DC1434 0130405 PR RT
M&I White DC456a 0120403 PR RT
MSM White DC781 0130416 PR RT
MSM Black DC1282a 0110527 PR RT
MSM White DC318a 0121010 PR RT
MSM Black DC1890 0111214 PR RT
UNK Black DC1875 0110805 PR RT
MSM Black DC395 0121226 PR RT
MSM Black DC1511 0120504 PR RT
MSM Black DC1563a 0120920 PR RT
MSM Black DC1717a 0110208 PR RT
MSM Black DC1738a 0140122 PR RT
MSM Black DC1812 0121226 PR RT
MSM Black DC1829a 0120203 PR RT
UNK Black DC497 0150220 PR RT
MSM Black DC1847 0130301 PR RT
MSM Black DC868 0140114 PR RT
MSM White DC1756 0110706 PR RT
MSM White DC484 0150306 PR RT
UNK Black DC932 0150113 PR RT
MSM Black DC894 0140402 PR RT
MSM Black DC733 0140310 PR RT
MSM Hispa DC746 0130311 PR RT
HRH Black DC1248 0121024 PR RT
HRH Black DC438 0141002 PR RT
MSM Black DC528a 0111020 PR RT
MSM Black DC1651a 0111028 PR RT
MSM Black DC1761a 0110916 PR RT
HRH Black DC523a 0111128 PR RT
UNK Black DC1084 0121222 PR RT
UNK Black DC472 0130212 PR RT
HRH Black DC1696a 0110304 PR RT
UNK Black DC130a 0110210 PR RT
HRH Black DC645a 0120501 PR RT
HRH Other DC620a 0120316 PR RT
HRH White DC1802 0110815 PR RT
HRH Black DC1707 0110804 PR RT
HRH Black DC949 0150324 PR RT
MSM Black DC1821 0120713 PR RT
HRH Black DC1740a 0110510 PR RT
UNK Black DC1133a 0110920 PR RT
MSM Black DC1473 0130410 PR RT
MSM Black DC505a 0130305 PR RT
HRH Black DC220a 0110518 PR RT
MSM Black DC148a 0150302 PR RT
HRH Black DC401a 0130625 PR RT
MSM Black DC1322 0130812 PR RT
UNK Black DC1175 0120907 PR RT
MSM Black DC1094 0120208 PR RT
MSM Black DC773 0130207 PR RT
HRH Black DC615 0120503 PR RT
M&I Black DC841 0121227 PR RT
MSM Black DC545 0111220 PR RT
MSM Black DC393 0110719 PR RT
MSM Black DC1412 0131107 PR RT
MSM Black DC1663a 0121227 PR RT
HRH Black DC782 0130401 PR RT
MSM Black DC1708 0130214 PR RT
MSM Black DC1241 0121019 PR RT
MSM Black DC687 0120927 PR RT
UNK Black DC249 0121211 PR RT
HRH Black DC904 0140611 PR RT
MSM Black DC1382 0110628 PR RT
MSM Other DC1152 0120821 PR RT
HRH Black DC1220a 0120816 PR RT
HRH Black DC1249a 0110322 PR RT

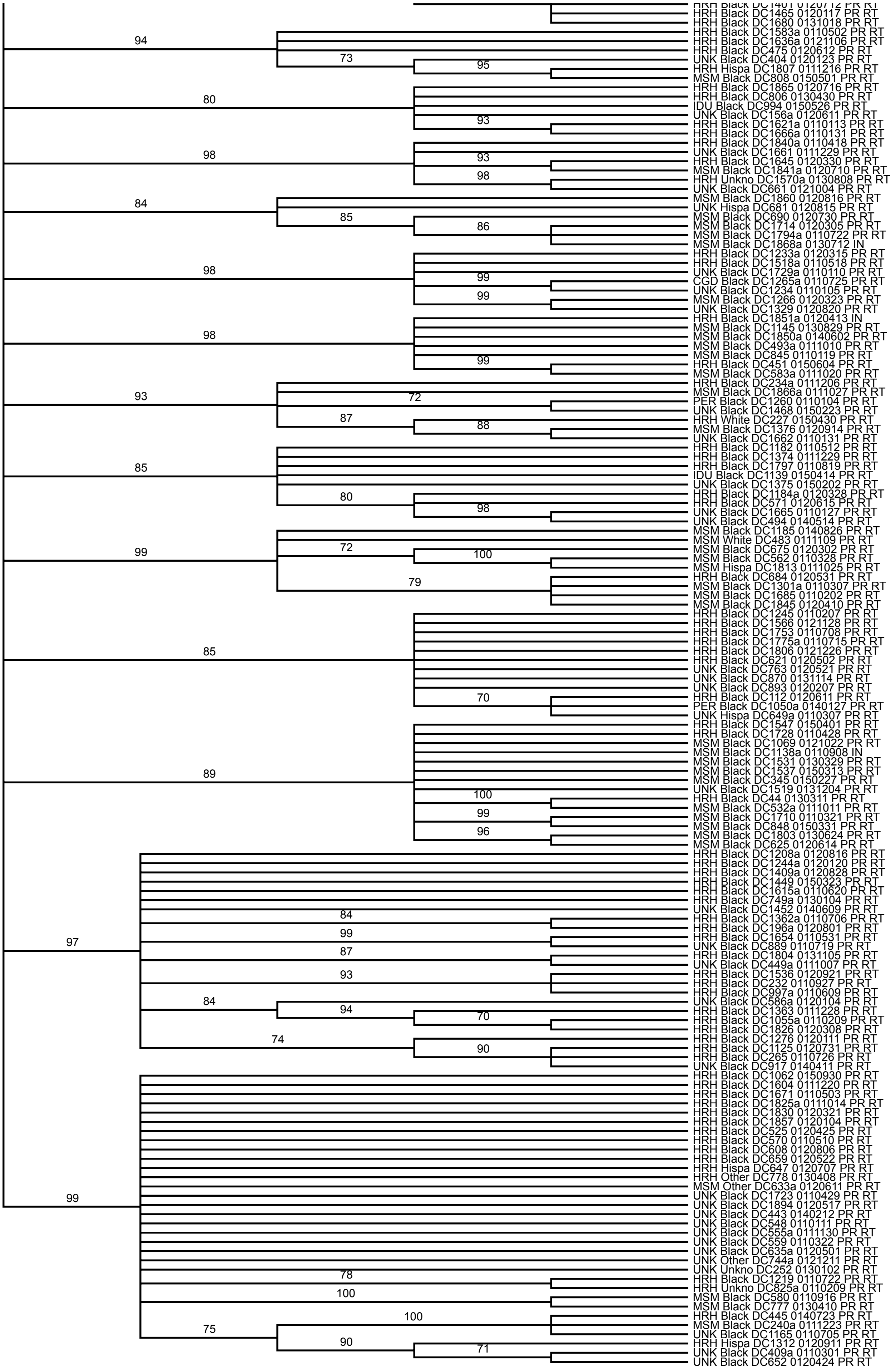
HRH Black DC1401 0120712 PR RT
HRH Black DC1465 0120117 PR RT
HRH Black DC1680 0131018 PR RT
94
HRH Black DC1583a 0110502 PR RT
HRH Black DC1636a 0121106 PR RT
HRH Black DC475 0120612 PR RT
73
UNK Black DC404 0120123 PR RT
95
HRH Hispa DC1807 0111216 PR RT
MSM Black DC808 0150501 PR RT
80
HRH Black DC1865 0120716 PR RT
HRH Black DC806 0130430 PR RT
IDU Black DC994 0150526 PR RT
UNK Black DC156a 0120611 PR RT
93
HRH Black DC1621a 0110113 PR RT
HRH Black DC1666a 0110131 PR RT
98
HRH Black DC1840a 0110418 PR RT
UNK Black DC1661 0111229 PR RT
93
HRH Black DC1645 0120330 PR RT
MSM Black DC1841a 0120710 PR RT
98
HRH Unkno DC1570a 0130808 PR RT
UNK Black DC661 0121004 PR RT
84
MSM Black DC1860 0120816 PR RT
UNK Hispa DC681 0120815 PR RT
85
MSM Black DC690 0120730 PR RT
86
MSM Black DC1714 0120305 PR RT
MSM Black DC1794a 0110722 PR RT
MSM Black DC1868a 0130712 IN
98
HRH Black DC1233a 0120315 PR RT
HRH Black DC1518a 0110518 PR RT
UNK Black DC1729a 0110110 PR RT
99
CGD Black DC1265a 0110725 PR RT
UNK Black DC1234 0110105 PR RT
99
MSM Black DC1266 0120323 PR RT
UNK Black DC1329 0120820 PR RT
98
HRH Black DC1851a 0120413 IN
MSM Black DC1145 0130829 PR RT
MSM Black DC1850a 0140602 PR RT
MSM Black DC493a 0111010 PR RT
MSM Black DC845 0110119 PR RT
99
HRH Black DC451 0150604 PR RT
MSM Black DC583a 0111020 PR RT
93
HRH Black DC234a 0111206 PR RT
MSM Black DC1866a 0111027 PR RT
72
PER Black DC1260 0110104 PR RT
UNK Black DC1468 0150223 PR RT
87
HRH White DC227 0150430 PR RT
88
MSM Black DC1376 0120914 PR RT
UNK Black DC1662 0110131 PR RT
85
HRH Black DC1182 0110512 PR RT
HRH Black DC1374 0111229 PR RT
HRH Black DC1797 0110819 PR RT
IDU Black DC1139 0150414 PR RT
UNK Black DC1375 0150202 PR RT
80
HRH Black DC1184a 0120328 PR RT
98
HRH Black DC571 0120615 PR RT
UNK Black DC1665 0110127 PR RT
UNK Black DC494 0140514 PR RT
99
MSM Black DC1185 0140826 PR RT
MSM White DC483 011109 PR RT
72
MSM Black DC675 0120302 PR RT
100
MSM Black DC562 0110328 PR RT
MSM Hispa DC1813 0111025 PR RT
79
HRH Black DC684 0120531 PR RT
MSM Black DC1301a 0110307 PR RT
MSM Black DC1685 0110202 PR RT
MSM Black DC1845 0120410 PR RT
85
HRH Black DC1245 0110207 PR RT
HRH Black DC1566 0121128 PR RT
HRH Black DC1753 0110708 PR RT
HRH Black DC1775a 0110715 PR RT
HRH Black DC1806 0121226 PR RT
HRH Black DC621 0120502 PR RT
UNK Black DC763 0120521 PR RT
UNK Black DC870 0131114 PR RT
UNK Black DC893 0120207 PR RT
70
HRH Black DC112 0120611 PR RT
PER Black DC1050a 0140127 PR RT
UNK Hispa DC649a 0110307 PR RT
89
HRH Black DC1547 0150401 PR RT
HRH Black DC1728 0110428 PR RT
MSM Black DC1069 0121022 PR RT
MSM Black DC1138a 0110908 IN
MSM Black DC1531 0130329 PR RT
MSM Black DC1537 0150313 PR RT
MSM Black DC345 0150227 PR RT
UNK Black DC1519 0131204 PR RT
100
HRH Black DC44 0130311 PR RT
MSM Black DC532a 0111011 PR RT
99
MSM Black DC1710 0110321 PR RT
MSM Black DC848 0150331 PR RT
96
MSM Black DC1803 0130624 PR RT
MSM Black DC625 0120614 PR RT
HRH Black DC1208a 0120816 PR RT
HRH Black DC1244a 0120120 PR RT
HRH Black DC1409a 0120828 PR RT
HRH Black DC1449 0150323 PR RT
HRH Black DC1615a 0110620 PR RT
HRH Black DC749a 0130104 PR RT
UNK Black DC1452 0140609 PR RT
84
HRH Black DC1362a 0110706 PR RT
HRH Black DC196a 0120801 PR RT
99
HRH Black DC1654 0110531 PR RT
UNK Black DC889 0110719 PR RT
97
87
HRH Black DC1804 0131105 PR RT
UNK Black DC449a 0111007 PR RT
93
HRH Black DC1536 0120921 PR RT
HRH Black DC232 0110927 PR RT
HRH Black DC997a 0110609 PR RT
84
UNK Black DC586a 0120104 PR RT
94
HRH Black DC1363 0111228 PR RT
70
HRH Black DC1055a 0110209 PR RT
HRH Black DC1826 0120308 PR RT
74
HRH Black DC1276 0120111 PR RT
90
HRH Black DC1125 0120731 PR RT
HRH Black DC265 0110726 PR RT
UNK Black DC917 0140411 PR RT
HRH Black DC1062 0150930 PR RT
HRH Black DC1604 0111220 PR RT
HRH Black DC1671 0110503 PR RT
HRH Black DC1825a 0111014 PR RT
HRH Black DC1830 0120321 PR RT
HRH Black DC1857 0120104 PR RT
HRH Black DC525 0120425 PR RT
HRH Black DC570 0110510 PR RT
HRH Black DC608 0120806 PR RT
HRH Black DC659 0120522 PR RT
HRH Hispa DC647 0120707 PR RT
HRH Other DC778 0130408 PR RT
MSM Other DC633a 0120611 PR RT
UNK Black DC1723 0110429 PR RT
99
UNK Black DC1894 0120517 PR RT
UNK Black DC443 0140212 PR RT
UNK Black DC548 0110111 PR RT
UNK Black DC555a 0111130 PR RT
UNK Black DC559 0110322 PR RT
UNK Black DC635a 0120501 PR RT
UNK Other DC744a 0121211 PR RT
UNK Unkno DC252 0130102 PR RT
78
HRH Black DC1219 0110722 PR RT
HRH Unkno DC825a 0110209 PR RT
100
MSM Black DC580 0110916 PR RT
MSM Black DC777 0130410 PR RT
100
HRH Black DC445 0140723 PR RT
75
MSM Black DC240a 0111223 PR RT
UNK Black DC1165 0110705 PR RT
90
HRH Hispa DC1312 0120911 PR RT
71
UNK Black DC409a 0110301 PR RT
UNK Black DC652 0120424 PR RT
