# Supplementary material for: Characterization of HIV diversity, phylodynamics and drug resistance in Washington, DC
Source: PLoS One. 2017 Sep 29;12(9):e0185644. doi: 10.1371/journal.pone.0185644 (PMC5621693; doi:10.1371/journal.pone.0185644)
Supplement: S2 Fig — (PDF) [file pone.0185644.s003.pdf]

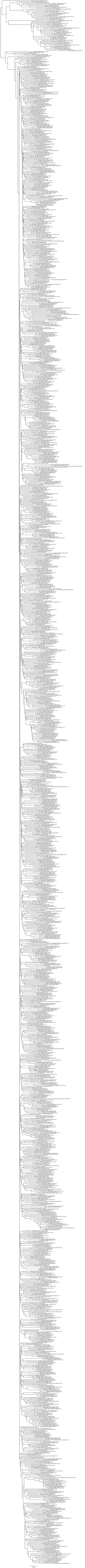

0.04
